# Hospitalizations from Hypertensive Diseases, Diabetes, and Arrhythmia in Relation to Low and High Temperatures: Population-Based Study

**DOI:** 10.1038/srep30283

**Published:** 2016-07-26

**Authors:** Li Bai, Qiongsi Li, Jun Wang, Eric Lavigne, Antonio Gasparrini, Ray Copes, Abderrahmane Yagouti, Richard T. Burnett, Mark S. Goldberg, Paul J. Villeneuve, Sabit Cakmak, Hong Chen

**Affiliations:** 1 Public Health Ontario, Toronto, ON, Canada; 2 Institute for Clinical Evaluative Sciences, Toronto, ON, Canada; 3 Air Health Science Division, Health Canada, Ottawa, ON, Canada; 4 School of Epidemiology, Public Health and Preventive Medicine, University of Ottawa, ON, Canada; 5 Department of Social and Environmental Health Research, London School of Hygiene and Tropical Medicine, London, UK; 6 Dalla Lana School of Public Health, University of Toronto, Toronto, ON, Canada; 7 Climate Change and Health Office, Health Canada, Ottawa, ON, Canada; 8 Population Studies Division, Health Canada, Ottawa, ON, Canada; 9 Department of Medicine, McGill University, Montreal, Quebec, Canada; 10 Division of Clinical Epidemiology, Research Institute of the McGill University Health Centre, Montreal, Quebec, Canada; 11 CHAIM Research Centre, Carleton University, Ottawa, ON, Canada

## Abstract

Little is known about the extent to which ambient temperatures contribute to the burden of hospitalizations from hypertensive diseases, diabetes, and arrhythmia. To fill this knowledge gap, we conducted a time-series study comprising entire population of Ontario, Canada during 1996–2013. A distributed lag non-linear model was developed to estimate the cumulative effect of temperatures over a 21-day lag period. We computed the burden of hospitalizations attributable to cold and heat. Furthermore, we separated the burden into components related to mild and extreme temperatures. Compared to the temperature with minimum risk of morbidity, cold temperatures (1^st^ percentile) were associated with a 37% (95% confidence interval: 5%, 78%) increase in hypertension-related hospitalizations whereas no significant association with hot temperatures (99^th^ percentile) was observed. Cold and hot temperatures were also associated with a 12% (1%, 24%) and a 30% (6%, 58%) increase in diabetes-related hospitalizations, respectively. Arrhythmia was not linked to temperatures. These estimates translate into ~10% of hypertension-related hospitalizations attributable to total cold, and ~9% from mild cold. Similarly, ~11% of diabetes-related hospitalizations were due to total heat, virtually all of which were from mild heat. In conclusion, ambient temperatures, especially in moderate ranges, contribute to excess hospitalizations from hypertension and diabetes.

Low and high ambient temperatures have been shown to increase mortality^1^, especially from cardiovascular-related causes^2^,^3^,^4^. A recent study comprising 13 countries with more than 74 million deaths between 1985 and 2012 estimated that 7.7% of mortality was attributable to temperature exposures^5^. There is also growing evidence that individuals living with cardiovascular diseases are likely to be at an increased risk of temperature-related mortality^1^. The physiological mechanisms underlying the cardiovascular effects of temperatures are not yet fully understood. However, high blood pressure, a leading risk factor of cardiovascular diseases, was found to occur more frequently in winter than in summer^6^,^7^,^8^. It is hypothesized that exposure to cold temperatures in winter may trigger subcutaneous vasoconstriction that can increase central blood volume, leading to higher blood pressure^9^, which may, in turn, damage and harden arteries, constrain blood supply to the heart, promote atherosclerosis, leading to cold-related cardiovascular morbidity^10^,^11^. In addition, it is postulated that summer-time high temperatures may also elevate blood pressure, impair the control of blood flow in the skin, weaken core temperature regulation, and heighten the risk of adverse cardiovascular events^12^,^13^.

To date, only one study has been conducted to investigate the impact of ambient temperatures on hospital admissions for hypertension, a condition characterized by a persistent elevation of the blood pressure^14^. Comprising all residents of New York City, US, this study reported that summer-time high temperatures were associated with reduced risk of hypertension-related hospital admissions, but it did not evaluate any possible effect from cold temperatures. The impact of temperatures on diabetes^15^,^16^,^17^ and arrhythmia^18^,^19^, two conditions that often co-exist^20^,^21^,^22^,^23^,^24^ with hypertension and share similar risk factors^25^, have been assessed in a handful of studies. A positive relationship between diabetes-related hospital admissions and apparent temperature (a composite index of temperature and humidity) was found in California, US^15^. The relationship between diabetes hospitalizations and heat waves was also investigated in two other studies, with a positive association reported in one study^16^ but null association in another^17^. More recently, two panel studies of patients implanted with cardioverter-defibrillator devices reported that cold temperatures may trigger arrhythmia^18^,^19^.

An estimated 30% of adults in the US^26^ and 22% in Canada^27^ have high blood pressure. In addition, diabetes affects 9.3% and 6.8% of the population in these two countries^28^,^29^, respectively. As well, more than 1.7% of Americans and 1.0% of Canadians are living with Atrial Fibrillation, the most common pathologic arrhythmia^30^,^31^. Given the considerable health burden of these three conditions and the important impact of cold and hot temperatures on triggering deaths from cardiovascular causes^5^, we conducted a study to investigate the associations between ambient temperatures and the risks of hospital admissions for hypertension, diabetes and arrhythmia in Ontario, Canada. In addition, we aimed to quantify the burden of hospital admissions from these conditions in association with cold and heat.

## Results

From 1 January 1996 to 31 December 2013, there were a total of 50,788 individuals who were admitted to a hospital due to hypertensive diseases, 324,034 due to diabetes, and 345,052 due to arrhythmia across Ontario (Table 1). At the time of admissions, the mean ages of patients were 64 years, 57 years, and 68 years old, respectively. Of them, ~16% of had congestive heart failure, ~13% had cancer, and 45% to 76% were recurrent admissions (depending on the outcome). The descriptive statistics of daily mean temperatures and relative humidity in each of the 14 health regions were reported in Supplementary Table S1. Overall, temperatures were warmer in the Southern and Central Ontario relative to those in the North.

**Figure 1:**
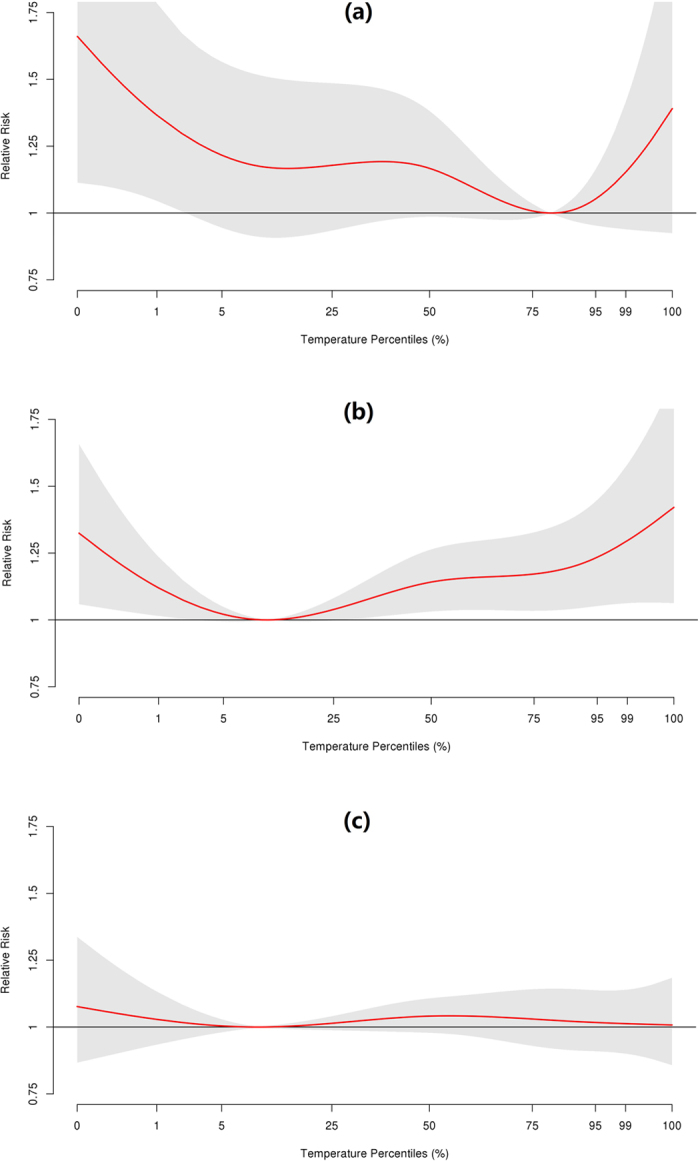
Cumulative exposure–response associations of daily mean temperatures and daily hospital admissions for (**a**) hypertension, (**b**) diabetes, and (**c**) arrhythmia over a lag of 21 days in Ontario, Canada, 1996–2013. The associations were modelled using natural cubic spline for temperature (4 *df*) with knots at the 10^th^, 50^th^ and 90^th^ percentiles of region-specific temperature distributions and using natural cubic for lags (3 *df*). The minimum morbidity temperature percentiles for hospital admissions for hypertension, diabetes and arrhythmia were 81^st^, 11^th^ and 10^th^, respectively.

Figure 1 shows overall cumulative exposure-response relationships between the three causes of hospitalizations and temperatures across a 21-day lag. We observed nonlinear V- or reverse J-shaped associations, with higher risks of hospitalizations at both extreme low and high temperatures for the three outcomes (with the exception of extreme high temperature with arrhythmia admissions). It is noteworthy that the three outcomes exhibited different MMP, with 81^st^ (18.55 °C) for hypertension, 11^th^ (−6.36 °C) for diabetes, and 10^th^ (−6.89 °C) for arrhythmia. We provided the exposure-response relationships between the three causes of hospitalizations and temperatures across a 21-day lag in four selected health regions in Supplementary Figs S1–S3.

Compared to the MMP, cold temperatures (at the 1^st^ percentile of temperature) were associated with a 37% (95% CI: 5%, 78%) increase in hospitalizations from hypertension, whereas no significant association was found with hot temperatures (at the 99^th^ percentile) (Table 2). In contrast, cold and hot temperatures were associated with a 12% (95% CI: 1%, 24%) and a 30% (95% CI: 6%, 58%) increases in hospitalizations from diabetes, respectively. In addition, compared the 1^st^ percentile of temperature to the 25^th^ percentile and the 99^th^ percentile temperature to the 75^th^ percentile, the effects of cold and heat on both outcomes remained elevated, but the effect sizes were more modest (Table 2). For arrhythmia, there was no strong evidence of cold and heat effects.

To quantify the temperature-related burden on hypertension and diabetes, we estimated the fractions and the numbers of hospitalizations from these two conditions that were attributable to cold and heat during the course of study (Table 3). For hypertension, we estimated that cold temperatures contributed to 9.99% (95% CI: 4.67, 13.29) of total hospitalizations (or 5,058 admissions) and hot temperatures contributed to 1.43% (95% CI: −1.02, 3.21) of hospitalizations. In contrast, 11.16% (95% CI: 7.50, 14.25) of hospitalizations from diabetes (36,045 admissions) were attributable to hot temperatures and 0.73% (−2.25, 2.83) of hospitalizations were due to cold temperatures.

In addition, we separated the burden into components related to mild and extreme temperatures, defined using cutoffs at 2.5^th^ and 97.5^th^ temperature percentiles. For hypertension, exposure to mild cold temperatures (temperatures from the MMP to the 2.5^th^ percentile) contributed to 9.28% (95% CI: 4.23, 12.57) of hospitalizations and only 0.72% (95% CI: 0.52, 0.86) was due to extreme cold temperatures (the 2.5^th^ percentile to the minimum temperature) (Table 3). For diabetes, mild hot temperatures (the MMP to the 97.5^th^ percentile temperature) contributed to 10.59% (95% CI: 7.07, 13.41) of hospitalizations and 0.58% (95% CI: 0.36, 0.70) was due to extreme hot temperatures (the 97.5^th^ percentile to the maximum temperature).

As well, we found that the risk of diabetes-related hospitalizations from heat was significantly higher in patients who had cancer (with cancer: RR = 1.42, 95% CI: 1.11, 1.82 vs. free of cancer: RR = 1.07, 95% CI: 0.94, 1.22) (*P*_interaction_ = 0.045) (see Supplementary Figs S4–S9). There was also a trend toward larger cold effects on increasing hypertension-related hospitalizations in patients who had diabetes (diabetics: RR = 1.70, 95% CI: 0.97, 2.99 vs. non-diabetics: RR = 1.03, 95% CI: 0.80, 1.32) (*P*_interaction_ = 0.11). Furthermore, elderly people who regularly took diuretics had a significantly higher risk for admissions from diabetes on hot days (RR: 1.27, 95% CI: 1.04, 1.55) than non-users (RR: 0.92, 95% CI: 0.75, 1.14) (*P*_interaction_ = 0.03).

Our estimates were insensitive to the change of degrees of freedom (*df*) for temperature from 4 to 6 and the change of the internal knot for lag from 1 to 2 (see Supplementary Table S2). Cumulative exposure-response associations over a lag of 14 days exhibited similar patterns but somewhat attenuated estimate of cold effect as compared to those over a lag of 21 days (see Supplementary Fig. S10). In addition, the risk estimates were not materially altered after adjusting for PM_2.5_ (see Supplementary Table S3).

## Discussion

In this population-based study in Ontario, we observed that low ambient temperature elevated the risk of hospitalization for hypertension, while increased hospital admissions for diabetes were associated with exposures to both low and high temperatures. We did not find strong evidence linking cold and heat to arrhythmia. In addition, we estimated that approximately 10% of hypertension hospitalizations were attributable to cold and 11% of diabetes admissions were attributable to heat. In contrast, 0.7% and 0.6% of hospitalizations for hypertension and diabetes were estimated to be attributable to extreme temperatures (extreme cold or heat).

To date, only one study investigated the association between ambient high temperature and hospitalizations for hypertension^14^. This study observed decreased risk of admissions related to heat, but did not examine possible effect from cold^14^. Several previous studies observed peaked diastolic and systolic blood pressure during winters^6^,^32^. Our study showed, for the first time, that cold exposure led to increased risk of hospitalization due to hypertension. The effect of cold temperature on hypertension-related hospitalizations may be mediated through pathways such as thermoregulation-mediated vasoconstriction, activation of hypothalamic-pituitary-adrenal axis and sympathetic nervous system, and sodium/volume retention and impaired endothelial-dependent vasodilation^9^ While we also observed a tendency for higher cold-related hospital admissions for hypertension among individuals with diabetes, this study lacks statistical power to detect significant heterogeneity. Given that approximately 71% of American adults and 63% of Canadian adults with diagnosed diabetes have concomitant hypertension^20^,^28^, further research on the temperature effects among patients living with both hypertension and diabetes is warranted.

Several studies suggest that overall adverse health impacts of winter cold in high-income countries may be decreasing in recent decades due to better housing and improved health care^33^,^34^. However, our observation of a strong cold effect on hypertension in Ontario showed that adverse effect of cold on cardiovascular conditions remained persistent in current days. This may be particularly relevant to low-income families and other economically vulnerable persons who are likely disproportionately exposed to cold temperatures in winters. Understanding the impact (and the pathway) of cold temperatures on health among potentially vulnerable subpopulations is important and requires further research.

Increased diabetes admissions were found to be associated with cold and hot temperatures in this study. Several previous studies have investigated the effects of high temperature on hospitalizations and emergency room visits due to diabetes^15^,^16^,^17^,^35^. In one study conducted in Sydney, Australia, hospital admissions for diabetes were positively associated with single-day heat extremes (odds ratio (OR): 1.16, 95% CI: 1.03, 1.30) and heat extremes lasting for a period of 3 consecutive days (OR:1.14, 95% CI: 1.01, 1.29)^16^. Similar results of immediate effects on both diabetes hospitalizations (% excess risk per 10°F: 4.0%, 95% CI: 1.9, 6.2) and emergency room visits (4.3%, 95% CI: 2.8, 5.9) were reported in California, US^15^,^35^. More recently, there was a report of no statistically significant association between diabetes hospitalizations and heat waves, defined as >2 consecutive extreme hot days in the US^17^. Our finding of cold effects on increasing diabetes admissions has not been previously reported. This finding has relevant clinical implications, as diabetic patients have been found to have higher hemoglobin A1c (HbA1c) levels over the winter months than during the warmer months^36^,^37^.

Less is known about the effect of temperature on hospital admissions for arrhythmia. Until recently, two studies found that among patients implanted with dual chamber implantable cardioverter-defibrillator devices, cold temperature increased episodes of ventricular arrhythmias^18^ and atrial fibrillation^19^ recorded by the devices. However, we did not find evidence on increased risk of arrhythmia hospitalizations associated with exposure to cold or heat in the present study. This inconsistent finding might be due to inherent different characteristics of study subjects, difference in outcome ascertainment and study designs, or chance.

Although the majority of studies on the association between health consequences and temperatures have focused on temperature extremes, we found that moderate but non-optimum temperatures appeared to pose a much greater impact on the burden of hospitalizations from ambient temperatures. We estimated that 9.3% of hypertension admissions were associated with exposure to cold temperatures in the moderate range, compared with temperature extremes (0.7%). In addition, moderate heat was responsible for 10.6% of diabetes hospitalizations, while only 0.6% of admissions were related to extreme heat. This suggests the need of improving public health programs and adaptive strategies by further considering the whole range of temperatures that include both mild and extreme temperatures. Our results also extend a recent report that 6.7% of temperature-attributable deaths occurred on moderately hot and moderately cold days, while extreme temperatures were only responsible for 0.9% of temperature-attributable deaths^5^. To our knowledge, the present analysis is the first study to date to quantify excess in hospital admissions from hypertension and diabetes due to low and high ambient temperatures.

Our analysis provides several additional insights into clinical practice and intervention planning. First, our results showed that hypertension and diabetes, two most common clinical conditions, have distinct responses to temperatures (minimum morbidity percentile: 81^st^ vs. 11^th^). This suggests that cold and heat may adversely affect the two conditions through different physiological processes. Clinicians should be aware of the different temperature tolerance among patients having the two conditions on cold and hot days. Specific intervention strategies aimed at persons living with certain medical conditions would be of great importance to reduce excess morbidity from exposures to cold and heat. For example, regularly monitoring blood pressure, enhancing medical management, and initiating health education programs that promote preventive behavioural change such as wearing proper clothes and averting physical exertion^38^,^39^ during the winter season may benefit those who have a history of high blood pressure. Second, we found that risks of hospitalizations for hypertension and diabetes could remain elevated for 21 days after exposure to cold/heat, implying that there is an opportunity to implement interventions and clinical treatments during this critical time window. Third, patients living with cancer tended to exhibit higher risk of heat-related hospitalizations for diabetes, likely explained by the possibility that radiation therapy, steroids and certain chemotherapy drugs may result in elevated blood glucose levels among diabetic patients^40^. Similarly, the elderly taking diuretics were found to be particularly vulnerable for diabetes-related hospitalizations as a result of heat. Taken together, these findings indicate the importance of considering susceptibility factors in implementing prevention and treatment of temperature health effects.

A strength of the study was the relatively long study period (17 years) with a large cohort of over 700,000 of hospital admissions. This study focused on entire population of Ontario, the most populous province in Canada, rather than selected cities. In addition, we used a flexible statistical approach^41^ to capture distributed lag and non-linear effects of temperatures on hospitalizations with adjustment for various potential confounders. The application of a recently developed multivariate meta-analytical model^42^ also allowed us to pool delayed and non-linear dependencies across Ontario. Furthermore, we collected data on several comorbidities and the use of anti-hypertensive medications using validated databases and we evaluated potential effect modification by these characteristics. Few previous studies reported the potential roles of medications in temperature-hospitalization associations.

Several limitations of the study should be noted. First, possible exposure misclassification may exist, because we were unable to measure personal exposure to temperatures. However, a previous study using both fixed-site monitoring data and spatial models to predict temperature at residence found similar associations between temperature and mortality^43^. Nevertheless, given the inherent imprecision of the spatially-derived exposure, our assessment of exposure was likely subject to nondifferential misclassification that may have attenuated the estimates^44^. Second, similar to previous studies on temperature and morbidity^45^, we considered only primary diagnosis as main reason for hospitalization. Although this allowed for more accurate ascertainment of health outcomes, it might have led to some underestimation of the burden on hospitalizations. Third, we did not include data on emergency department visits; our results thus could not capture the effects of temperature on the events of the three conditions that were more acute but were not severe enough for hospital admissions. Lastly, we did not control for PM_2.5_ in the main analyses due to substantial missing data. However, in the sensitivity analyses, we found that adjusting for PM_2.5_ did not materially change the risk estimates when we restricted the analyses to the study period with available PM_2.5_ data.

Our findings add weight to a growing body of literature on the health effects of ambient temperatures, and extend existing literature on increased risks of hospitalization for hypertension, diabetes and arrhythmia in relation to temperature exposures. We also show that mild but non-optimum temperatures contributed to much greater burden of hospital admissions for hypertension and diabetes than that from temperature extremes. Therefore, the full range of temperature effects, including both extreme and mild temperature contributions, should be taken into account in order to further reduce the adverse effects of ambient cold and heat.

## Methods

### Study Population and Hospitalization Data

The study population included all residents of Ontario who were admitted to a hospital in Ontario between January 1, 1996 and December 31, 2013 with a primary or most responsible diagnosis of hypertensive diseases (International Classification of Disease, 9^th^ Revision [ICD-9] codes 401-405 and 10^th^ Revision [ICD-10] codes I10-13, I15), diabetes (ICD-9 code 250 and ICD-10 codes E10-E14) or arrhythmia (ICD-9 code 427; ICD-10 codes I46.0, I46.9, I47-I49, R00.1). Using the Hospital Discharge Abstract Database of the Canadian Institute for Health Information (CIHI), we obtained all hospital admissions in Ontario including detailed diagnostic and procedural information^46^. We excluded patients transferred from other acute-care hospitals. Ontario is the most populous province in Canada, consisting of nearly 40% of Canadians (~14 millions in 2015)^47^. The healthcare system of Ontario is organized into 14 health regions (known as Local Health Integration Networks) that are responsible for planning, integrating, and funding various local health care services. Because of single-payer healthcare system, the CIHI Discharge Abstract Database captures entire population in Ontario.

We obtained the 6-character alphanumeric postal codes for all patients’ places of residence prior to their hospitalizations using data linkage to the Registered Persons Database, a registry of all Ontario residents who have ever had a valid health card. Data linkage was performed through individuals’ unique encrypted health card numbers. Consistent with previous studies of short-term effects of air pollution on morbidity in Ontario^48^, we calculated daily counts of hospital admissions occurring in each of the 14 health regions in Ontario for each of the three conditions, using patients’ postal code addresses. This will allow for creating tailored interventions by regions because health budgets are allocated by health regions in Ontario. The study was approved by the Research Ethics Board of the University of Toronto and was carried out in accordance with the approved guidelines (Protocol reference: 28527).

### Meteorological Data

Hourly weather data were obtained from Environment Canada at 125 monitoring stations across Ontario (median distance between a residence and the nearest weather station is 12 kilometers, with interquartile range of 8 to 28 kilometers, depending on the health regions) (see Supplementary Table S1 for more details). The weather data included hourly temperatures (°C) and relative humidity for each day during the study period^49^. We computed daily mean, maximum, minimum, and standard deviation of each meteorological variable for each station, and then averaged daily data across all weather stations within each health region. Consistent with previous studies^5^,^50^, we used mean temperature to assess temperature effects in this analysis, because it represents the exposure throughout the entire day and night, and it was shown to be a better predictor of mortality than other measures of temperature in our previous study^2^. The health region-specific mean daily estimates were then assigned to study subjects using their postal code residences before hospital admissions.

### Potential Confounding Variables

Daily average concentrations of air pollutants including nitrogen dioxide (NO_2_), ozone (O_3_) and fine particulate matter (<2.5 μm in aerodynamic diameter, PM_2.5_) were derived from air quality monitoring stations in Ontario (a total of 60 stations in 2013). For NO_2_ and O_3_, we calculated health region-wide average daily estimates. Due to a considerable number of missing values, we did not consider PM_2.5_ in main analyses, but assessed its potential role in sensitivity analysis. To account for potential confounding by influenza^51^, we obtained in each health region the daily number of physician office visits due to influenza. We also created indicator variables for statutory holidays and the day of week to control for potential “holiday effects” and weekly pattern.

### Potential Effect Modifiers

From the CIHI Discharge Abstract Database, we extracted each patient’s date of birth and sex. In addition, to investigate whether people with comorbid conditions were at increased risk of hospitalizations for exposure to cold and heat, we *a prior* ascertained seven comorbid conditions, including: diabetes; hypertension; arrhythmia; congestive heart failure; disorders of fluid, electrolyte, and acid-base balance; renal failure; and cancer (ICD-9 and ICD-10 diagnosis codes were listed in Supplementary Table S4)^35^,^52^. using the records of hospitalizations from these conditions (based on all diagnostic codes) within five years before the hospitalizations under investigation. Furthermore, we assessed the potential effect modification by the intake of diuretics, ACE inhibitors, beta blockers or calcium channel blockers, four common medications used to treat hypertensive diseases, within 90 days prior to hospital admissions. Previous studies showed that the use of anti-hypertensive medications may reduce the body’s thermoregulatory capacity and heat tolerance, which may, in turn, result in a dangerously high core temperature in hot ambient conditions^53^,^54^. Because publicly funded prescription drugs are available to people aged 65 and above, the data obtained from the Ontario Drug Benefits database contained claims for prescription for a subset of the study population.

### Statistical Analysis

Our analysis was performed in two stages. In the first stage, we assessed the estimate of the association between daily mean temperatures and daily hospital admissions for each condition in each health region, reported as relative risk (RR), using a distributed lag non-linear model (DLNM). Consistent with previous studies^55^, in all models, we adjusted for seasonality and long-term trends using natural cubic spline with 7 *df* per year for time. In addition, we controlled for relative humidity, NO_2_, O_3_, statutory holidays, a day-of-week indicator, and daily influenza activity. Consistent with previous multi-region studies of temperature effects^5^,^56^, we modeled the exposure-response association using natural cubic spline for temperature (4 *df*) with knots at the 10^th^, 50^th^ and 90^th^ percentiles of health region-specific temperature distributions and using natural cubic for lags (3 *df*) with internal knots placed at equally-spaced values in the log scale of lags. This choice of model was found to lead to a significantly better fit than other models with different *df* and knot locations (see Supplementary Table S5). We evaluated the model fits using Akaike’s Information Criterion for quasi-Poisson (Q-AIC)^57^. In all the models, we used a 21-day lag period to capture the cumulative temperature effects on hospitalizations^5^.

In the second stage of our analyses, we applied a multivariate meta-analysis^42^ to derive the pooled estimates across Ontario. For each condition, we first identified the lowest point of the Ontario-wide cumulative exposure-response curve as the minimum morbidity temperature (MMT), corresponding to a minimum morbidity percentile (MMP) between the 1^st^ and the 99^th^ percentiles^5^,^56^. The Ontario-wide exposure-response association was then modeled again using the MMT as the reference value. Consistent with previous studies, we estimated the cumulative effects of cold over a 21-day lag by calculating the risk of hospitalization at the 1^st^ percentile of temperature relative to the 25^th^ percentile of temperature and to the MMP, respectively^52^,^58^. Similarly, we estimated heat effects at the 99^th^ percentile of temperature relative to the 75^th^ percentile of temperature and the MMP, respectively^52^,^58^.

To quantify the burden of hospitalizations due to temperature exposures, we calculated the fractions and the numbers of excess hospitalizations attributable to high and low temperatures, respectively. Consistent with previous studies, we used the fitted meta-analytical model to produce the best linear unbiased prediction of the overall cumulative exposure-response association in each of 14 health regions, using a recently developed method^5^,^42^,^59^. This approach allows to obtain more precise exposure-response association in areas with small daily hospitalization counts by borrowing information from areas with larger populations sharing similar characteristics^42^. We derived the MMT/MMP from the overall cumulative exposure-response association, and referred to the temperature value as the optimum temperature^5^,^59^. The total attributable number of hospitalizations due to non-optimum temperatures was calculated by summing the contributions from all the days during the study period^5^,^59^. The total attributable fraction was calculated by dividing the total attributable number of hospitalizations by the total number of hospitalizations, and multiplying by 100. We also calculated the components attributable to cold and heat by summing the contributions from days with temperatures lower or higher than the optimum temperature, respectively. Furthermore, we separated the cold- and heat-related burden into components attributable to mild and extreme temperatures. We defined extreme cold and heat as temperatures from the minimum to the 2.5^th^ temperature percentile and from the maximum to the 97.5^th^ percentile^5^,^59^. Mild temperatures were defined as the ranges between the optimum temperature and the two above mentioned cutoffs. The empirical confidential intervals (CIs) for attributable fractions were estimated using Monte Carlo simulations^5^,^59^.

To identify factors that may modify the effects of cold and heat on hospitalizations, we conducted stratified analyses by age (<65 years vs. ≥65 years), gender and the presence of seven selected preexisting comorbidities (diabetes, hypertension, arrhythmia, congestive heart failure, disorders of fluid, electrolyte, and acid-base balance, renal failure and cancer). We also assessed potential effect modification by the intake of the four types of medications (diuretics, ACE inhibitors, beta blockers and calcium channel blockers) among those aged 65 and above. We used a multivariate Wald test to assess the heterogeneity of the coefficients from each of these categories^60^.

To test the robustness of our results, we conducted various sensitivity analyses by changing the *df* for temperature from 4 to 6, the internal knot for lag from 1 to 2, and reducing the lag period from 21 days to 14 days. Additionally, we adjusted daily concentration of PM_2.5_ (available from 2003–2010) in a subset of the population. Statistical analyses were conducted using the R software (version 3.0.3) with the “dlnm” package and the “mvmeta” package.

## Additional Information

**How to cite this article**: Bai, L. *et al.* Hospitalizations from Hypertensive Diseases, Diabetes, and Arrhythmia in Relation to Low and High Temperatures: Population-Based Study. *Sci. Rep.* **6**, 30283; doi: 10.1038/srep30283 (2016).

## Supplementary Material

Supplementary Information

## Figures and Tables

**Table 1:** Descriptive statistics of study population in Ontario, Canada, 1996–2013.

Characteristics	Cause-specific Hospitalizations^a^		
	Hypertension N = 50,788	Diabetes N = 324,034	Arrhythmia N = 345,052
Demographic characteristics			
Age, mean (IQR), year	64.2 (25)	57.3 (26)	68.3 (20)
Men (%)	44.1	55.7	53.1
Comorbidity (%)			
Hypertension	47.5^b^	31.7	28.2
Diabetes	19.4	75.5^b^	14.8
Arrhythmia	12.8	10.4	45.3^b^
Congestive heart failure	15.5	14.8	17.0
Disorders of fluid, electrolyte, and acid-base balance	14.1	17.9	8.0
Renal diseases	24.4	16.4	6.4
Cancer	13.3	11.5	15.2

^a^Hospitalizations were identified using hospital discharge data.

^b^These statistics indicate recurring hospitalizations for hypertension, diabetes and arrhythmia. IQR: interquartile range.

**Table 2:** Cumulative relative risks (RRs) and 95% confidence interval (CIs) for cold and heat effects on daily hospitalizations for hypertension, diabetes and arrhythmia over a lag of 21 days in Ontario, Canada, 1996–2013.

	Cause-specific Hospitalizations		
	Hypertension	Diabetes	Arrhythmia
Cold effect^a^			
1^st^ vs. 25^th^	1.16 (0.94, 1.43)	1.08 (0.97, 1.19)	1.01 (0.92, 1.12)
1^st^ vs. MMP^b^	1.37 (1.05, 1.78)^*^	1.12 (1.01, 1.24)^*^	1.03 (0.93, 1.13)
Heat effect^c^			
99^th^ vs. 75^th^	1.14 (0.92, 1.43)	1.11 (0.98, 1.25)	0.98 (0.91, 1.07)
99^th^ vs. MMP	1.15 (0.94, 1.42)	1.30 (1.06, 1.58)^*^	1.01 (0.90, 1.14)

^a^Cold effects were examined by calculating relative risks associated with the 1^st^ percentile of temperature relative to the 25^th^ percentile of temperature and relative to MMP.

^b^The MMP for hospital admissions for hypertension, diabetes and arrhythmia were 81^st^, 11^th^ and 10^th^, respectively. The corresponding minimum morbidity temperature values (°C) were 18.55, −6.36 and −6.89, respectively.

^c^Heat effects were examined by calculating relative risks associated with the 99^th^ percentile of temperature relative to the 75^th^ percentile of temperature and relative to MMP. MMP: the minimum morbidity temperature percentile; ^*^P < 0.05.

**Table 3:** Estimated attributable fractions (%), attributable numbers, and 95% empirical confidence intervals (CIs) for cold and heat effects on daily hospitalizations due to hypertension and diabetes over a lag of 21 days in Ontario, Canada, 1996–2013.

	Hypertension		Diabetes	
	Attributable fraction (%)	Attributable number	Attributable fraction (%)	Attributable number
Total cold^a^	9.99% (4.67, 13.29)^*^	5,058 (2,362, 6,726)^*^	0.73% (−2.25, 2.83)	2,367 (−7,280, 9,134)
Extreme cold^b^	0.72% (0.52, 0.86)^*^	363 (261, 436)^*^	0.32% (0.16, 0.44)^*^	1,029 (513, 1,409)^*^
Mild cold^c^	9.28% (4.23, 12.57)^*^	4,697 (2,139, 6,360)^*^	0.42% (−2.01, 2.27)	1,341 (−6,481, 7,338)
Total heat^a^	1.43% (−1.02, 3.21)	726 (−514, 1,625)	11.16% (7.50, 14.25)^*^	36,045 (24,209, 46,024)^*^
Extreme heat^b^	0.31% (0.13, 0.45)^*^	158 (65, 226)^*^	0.58% (0.36, 0.70)^*^	1,860 (1,164, 2,271)^*^
Mild heat^c^	1.12% (−1.03, 2.80)	569 (−524, 1,417)	10.59% (7.07, 13.41)^*^	34,200 (22,840, 43,290)^*^

^a^Total burden of hospitalizations is the sum of extreme and mild contributions.

^b^Extreme cold was defined as temperatures lower than the 2.5^th^ percentile while extreme heat was defined as temperatures higher than the 97.5^th^ percentile.

^c^Mild cold was defined as temperatures in the range between the temperature with minimum hospitalization risk (referred to as optimal temperature) and the 2.5^th^ percentile while mild heat was defined as temperatures in the range between the optimum temperature and the 97.5^th^ percentile; ^*^P < 0.05.
