# Supplementary material for: Hospitalizations from Hypertensive Diseases, Diabetes, and Arrhythmia in Relation to Low and High Temperatures: Population-Based Study
Source: Sci Rep. 2016 Jul 26;6:30283. doi: 10.1038/srep30283 (PMC4960559; doi:10.1038/srep30283)
Supplement: Supplementary Information [file srep30283-s1.doc]

**Supplemental Material**

**Hospitalizations from Hypertensive Diseases, Diabetes, and Arrhythmia in Relation to Low and High Temperatures: Population-Based Study**

Li Bai,^1,2^ Qiongsi Li,^1^ Jun Wang,^1^ Eric Lavigne,^3,4^ Antonio Gasparrini,^5^ Ray Copes,^1,6^ Abderrahmane Yagouti,^7^ Richard T. Burnett,^8^ Mark S. Goldberg,^9,10^ Paul J. Villeneuve,^6,11^ Sabit Cakmak^8^ & Hong Chen^1,2,6*^

^1^ Public Health Ontario, Toronto, ON, Canada

^2^ Institute for Clinical Evaluative Sciences, Toronto, ON, Canada

^3^ Air Health Science Division, Health Canada, Ottawa, ON, Canada

^4^ School of Epidemiology, Public Health and Preventive Medicine, University of Ottawa, ON, Canada

^5^ Department of Social and Environmental Health Research, London School of Hygiene and Tropical Medicine, London, UK

^6^ Dalla Lana School of Public Health, University of Toronto, Toronto, ON, Canada

^7^ Climate Change and Health Office, Health Canada, Ottawa, ON, Canada

^8^ Population Studies Division, Health Canada, Ottawa, ON, Canada

^9^ Department of Medicine, McGill University, Montreal, Quebec, Canada

^10^ Division of Clinical Epidemiology, Research Institute of the McGill University Health Centre, Montreal, Quebec, Canada

^11^ CHAIM Research Centre, Carleton University, Ottawa, ON, Canada

**^*^Correspondence:**

Hong Chen, PhD

Public Health Ontario

480 University Avenue, Suite 300

Toronto, Ontario M5G 1V2

Tel: 647-260-7109

Email: hong.chen@oahpp.ca

**Table S1.** Descriptive statistics of weather data in Ontario, Canada during 1996-2013

| **Region** | **The number of weather monitoring stations** | **Mean humidity (%)** | **Temperature** | | | | | | | | | |
| --- | --- | --- | --- | --- | --- | --- | --- | --- | --- | --- | --- | --- |
|  |  |  | **Mean** | **Min** | **1^st^** | **10^th^** | **25^th^** | **Med** | **75^th^** | **90^th^** | **99^th^** | **Max** |
| Ontario | 125 | 74.0 | 7.6 | -22.9 | -16.0 | -6.9 | -0.6 | 8.0 | 17.0 | 20.9 | 25.2 | 29.3 |
| Health regions |  |  |  |  |  |  |  |  |  |  |  |  |
| 1. Erie St. Clair | 9 | 74.9 | 9.7 | -19.3 | -11.7 | -3.8 | 1.4 | 10 | 18.7 | 22.5 | 26.4 | 30.2 |
| 2. South West | 6 | 76.1 | 8.1 | -19.6 | -13.5 | -5.7 | 0.0 | 8.4 | 17.0 | 20.7 | 24.8 | 28.6 |
| 3. Waterloo Wellington | 2 | 76.1 | 7.8 | -22.3 | -15.1 | -6.4 | -0.4 | 8.2 | 16.9 | 20.9 | 25.7 | 29.8 |
| 4. Hamilton Niagara Haldimand Brant | 9 | 75.0 | 9.1 | -18.2 | -12.3 | -4.3 | 1.2 | 9.3 | 18.2 | 22 | 26.2 | 30.1 |
| 5. Central West | 4 | 71.9 | 8.5 | -20.9 | -14.2 | -5.6 | 0.4 | 8.8 | 17.8 | 21.8 | 26.5 | 31.0 |
| 6. Mississauga Halton | 1 | 69.6 | 9.4 | -17.9 | -11.4 | -3.5 | 1.8 | 9.3 | 18.2 | 22.2 | 26.8 | 31.2 |
| 7. Toronto Central | 3 | 73.1 | 9.2 | -19.3 | -12.1 | -3.6 | 1.9 | 9.1 | 17.9 | 21.7 | 25.7 | 30.5 |
| 8. Central | 1 | 70.8 | 8.4 | -21.9 | -14.8 | -5.8 | 0.2 | 8.6 | 17.7 | 21.7 | 26.8 | 32.2 |
| 9. Central East | 6 | 74.7 | 7.5 | -23.7 | -16.3 | -6.2 | 0.1 | 7.8 | 16.4 | 20.1 | 24.0 | 29.3 |
| 10. South East | 7 | 75.4 | 7.7 | -24.9 | -16.9 | -6.9 | -0.2 | 8.2 | 17.1 | 21.0 | 24.8 | 28.6 |
| 11. Champlain | 3 | 71.8 | 6.9 | -26.8 | -19.6 | -9.5 | -1.9 | 7.7 | 17.2 | 21.2 | 25.8 | 29.8 |
| 12. North Simcoe Muskoka | 9 | 77.2 | 7.0 | -24.7 | -17.7 | -7.5 | -1.0 | 7.4 | 16.4 | 20.2 | 24.7 | 27.4 |
| 13. North East | 31 | 74.4 | 4.6 | -28.4 | -22.0 | -11.7 | -4.3 | 5.4 | 14.9 | 18.8 | 22.7 | 26.2 |
| 14. North West | 34 | 75.0 | 2.2 | -33.1 | -25.8 | -16.1 | -7.4 | 3.4 | 13.4 | 17.6 | 21.8 | 24.8 |

**Table S2.** Cumulative relative risks (RRs) and 95% confidence interval (CIs) for the cold and heat effects on daily hospitalizations for hypertension, diabetes and arrhythmia over 21 lag days, using different degrees of freedom (*df*) for temperature and lag

| ***Df* for temperature** | **Cold effects^a^** | |  | **Heat effects^b^** | |
| --- | --- | --- | --- | --- | --- |
|  | 1 internal knot for lag | 2 internal knots for lag |  | 1 internal knot for lag | 2 internal knots for lag |
| **Hypertension** |  |  |  |  |  |
| 4 *df* | 1.16 (0.94, 1.43) | 1.15 (0.93, 1.42) |  | 1.14 (0.92, 1.43) | 1.16 (0.93, 1.45) |
| 5 *df* | 1.17 (0.94, 1.47) | ― |  | 1.11 (0.88, 1.39) | ― |
| 6 *df* | 1.19 (0.95, 1.49) | ― |  | 1.11 (0.87, 1.40) | ― |
| **Diabetes** |  |  |  |  |  |
| 4 *df* | 1.08 (0.97, 1.19) | 1.08 (0.97, 1.20) |  | 1.11 (0.98, 1.25) | 1.12 (0.99, 1.26) |
| 5 *df* | 1.10 (1.00, 1.22) | ― |  | 1.07 (0.94, 1.21) | ― |
| 6 *df* | 1.10 (0.99, 1.22) | ― |  | 1.07 (0.94, 1.22) | ― |
| **Arrhythmia** |  |  |  |  |  |
| 4 *df* | 1.01 (0.92, 1.12) | 1.01 (0.92, 1.12) |  | 0.98 (0.91, 1.07) | 0.98 (0.90, 1.07) |
| 5 *df* | 1.03 (0.93, 1.14) | ― |  | 0.95 (0.88, 1.04) | ― |
| 6 *df* | 1.04 (0.94, 1.15) | ― |  | 0.97 (0.89, 1.05) | ― |

^a^ Cold effects were examined by calculating relative risks associated with the 1^st^ percentile of temperature relative to the 25^th^ percentile of temperature. ^b^Heat effects were examined by calculating relative risks associated with the 99^th^ percentile of temperature relative to the 75^th^ percentile of temperature.

**Table S3.** Cumulative relative risks (RRs) and 95% confidence interval (CIs) for the cold and heat effects on daily hospitalizations for hypertension, diabetes and arrhythmia over 21 lag days, with and without adjusting for ambient fine particles (PM_2.5_)^*^.

|  | **Cold effects^a^** | |  | **Heat effects^b^** | |
| --- | --- | --- | --- | --- | --- |
|  | Adjusting for PM_2.5_ | Not adjusting for PM_2.5_ |  | Adjusting for PM_2.5_ | Not adjusting for PM_2.5_ |
| Hypertension | 1.11 (0.84, 1.48) | 1.12 (0.84, 148) |  | 1.10 (0.79, 1.52) | 1.09 (0.78, 1.51) |
| Diabetes | 1.00 (0.87, 1.15) | 1.00 (0.87, 1.15) |  | 1.11 (0.92, 1.33) | 1.11 (0.92, 1.33) |
| Arrhythmia | 0.99 (0.88, 1.13) | 0.99 (0.87, 1.12) |  | 0.96 (0.86, 1.08) | 0.97 (0.86, 1.08) |

^*^This analysis was restricted to the period from 2003 to 2010 when the data of PM_2.5_ was available. ^a^Cold effects were examined by calculating relative risks associated with the 1^st^ percentile of temperature relative to the 25^th^ percentile of temperature. ^b^Heat effects were examined by calculating relative risks associated with the 99^th^ percentile of temperature relative to the 75^th^ percentile of temperature.

**Table S4. International Classification of Diseases (ICD) codes of four comorbidities.**

|  | **ICD-9** | **ICD-10** |
| --- | --- | --- |
| Congestive heart failure | 428 | I50 |
| Disorders of fluid, electrolyte, and acid-base balance | 276 | E86-E87 |
| Renal failure | 584–588 | N17-N19 |
| Cancer | 140-239 | C00-D48 |

**Table S5.** Comparison of Akaike Information Criteria for quasi-Poisson (Q-AIC) values for the relationship between temperature and hospitalization in Ontario, using distributed lag non-linear models**^*^.**

|  | **Natural cubic spline for temperature**  **with three different knot locations** | | |
| --- | --- | --- | --- |
|  | 10^th^, 50^th^ and 90^th^ | 10^th^, 75^th^ and 90^th^ | 10^th^, 35^th^, 65^th^ and 90^th^ |
| Hypertension | 146840.94 | 146843.13 | 146882.79 |
| Diabetes | 327352.31 | 327364.90 | 327354.51 |
| Arrhythmia | 311383.65 | 311390.97 | 311418.64 |

**^*^**Models were fitted using natural cubic for lags (3 degrees of freedom) with three internal knots placed at equally-spaced values in the log scale of lags and adjusting for seasonality and long-term trend using natural cubic spline for time (7 degrees of freedom per year), relative humidity, nitrogen dioxide, ozone, statutory holidays, a day-of-week indicator, and daily influenza activity.


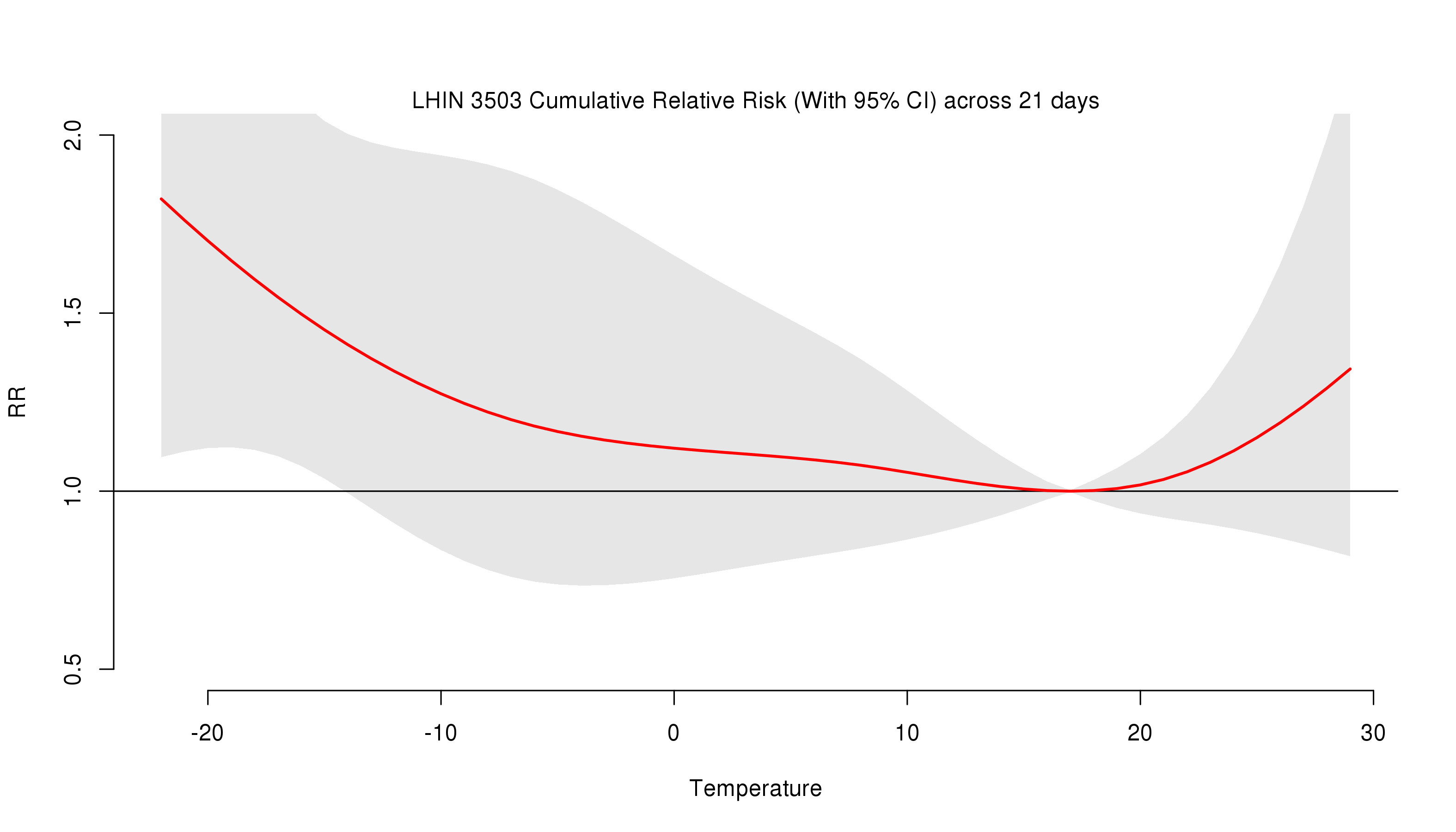

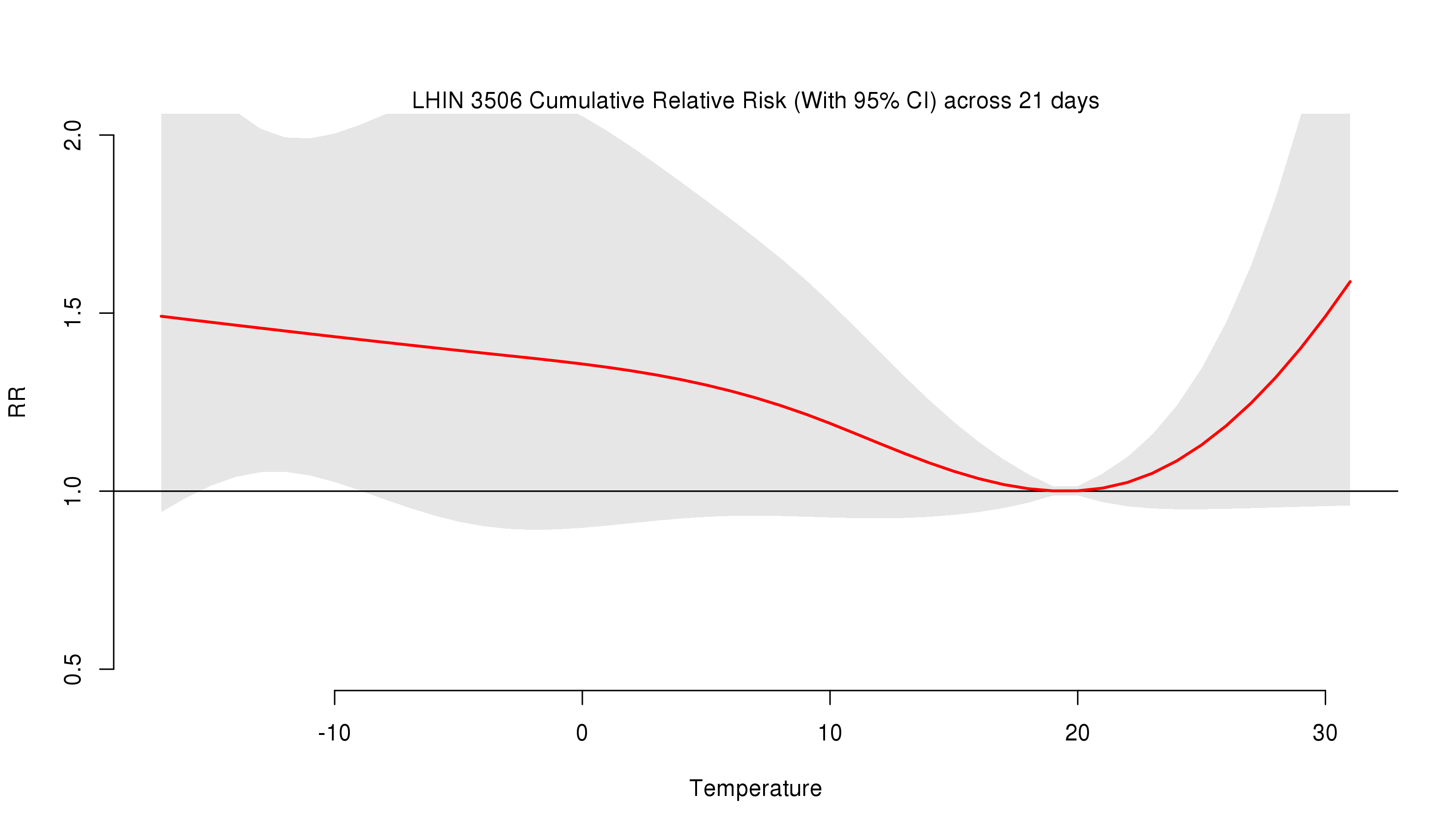


**(a)**

**(b)**


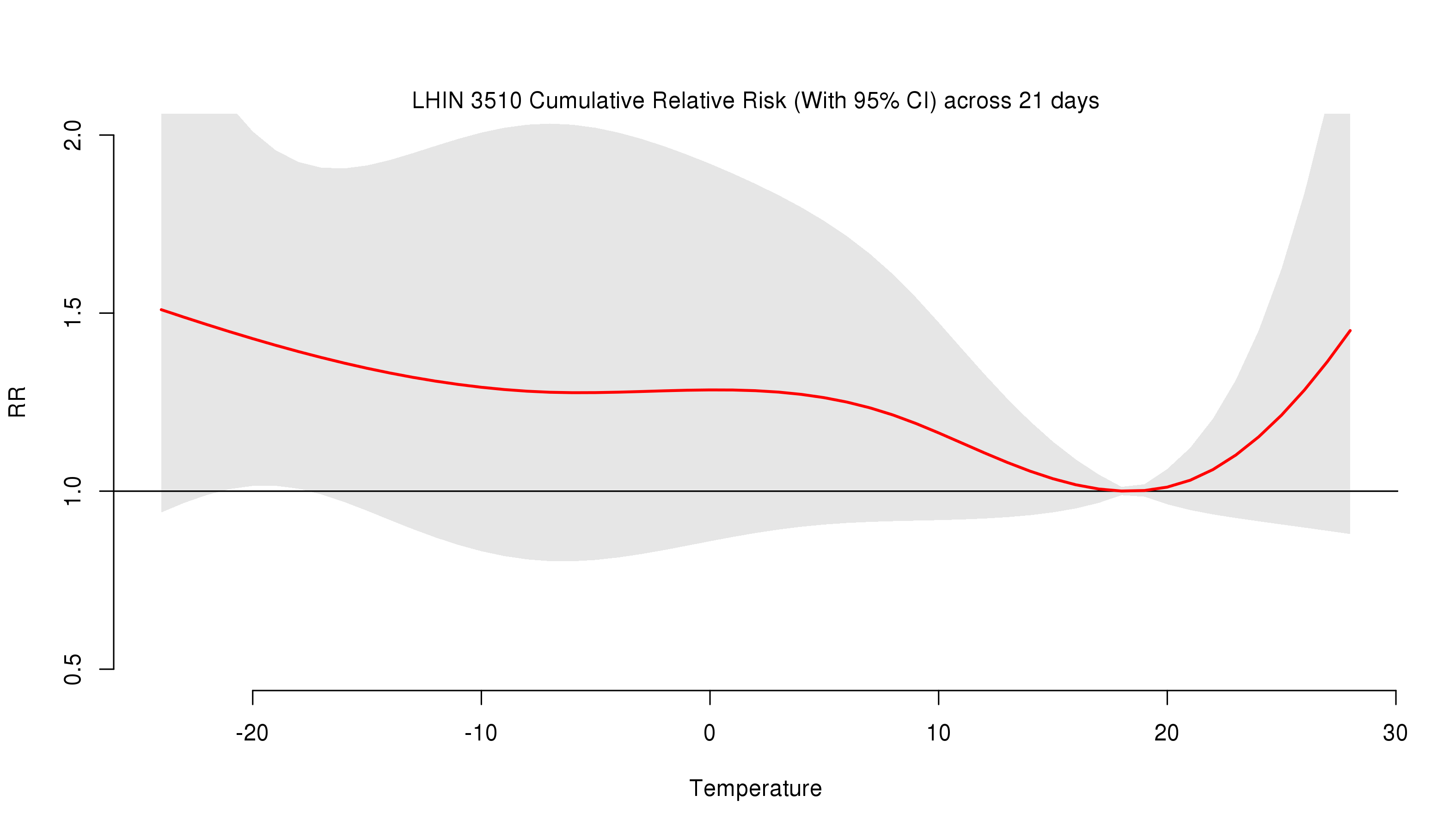

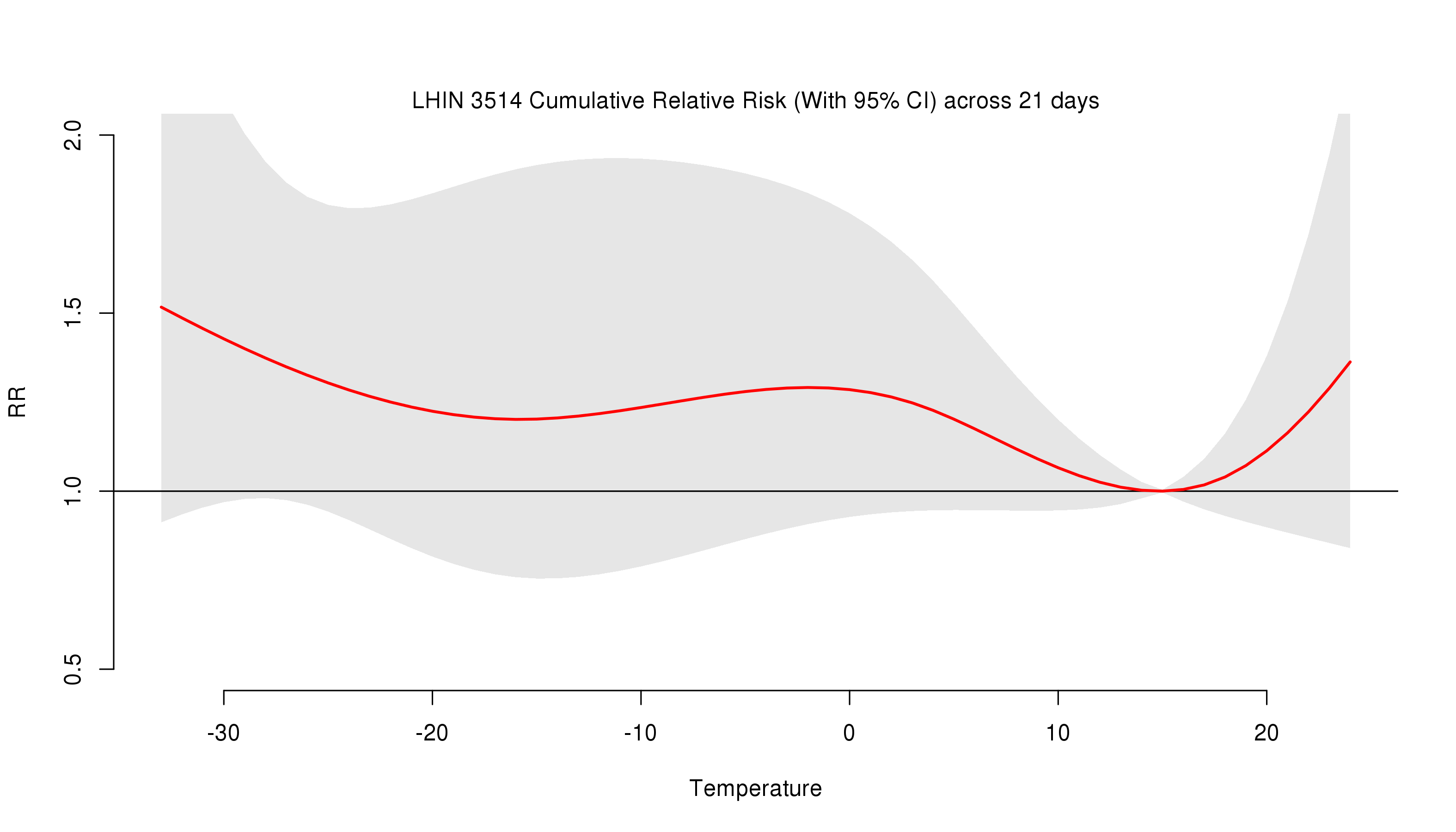


**(c)**

**(d)**

**Figure S1. Cumulative exposure–response associations of daily mean temperatures and daily hospital admissions for hypertension over a lag of 21 days in (a) Waterloo Wellington, (b) Mississauga Halton, (c) South East and (d) North West, 1996-2013.**


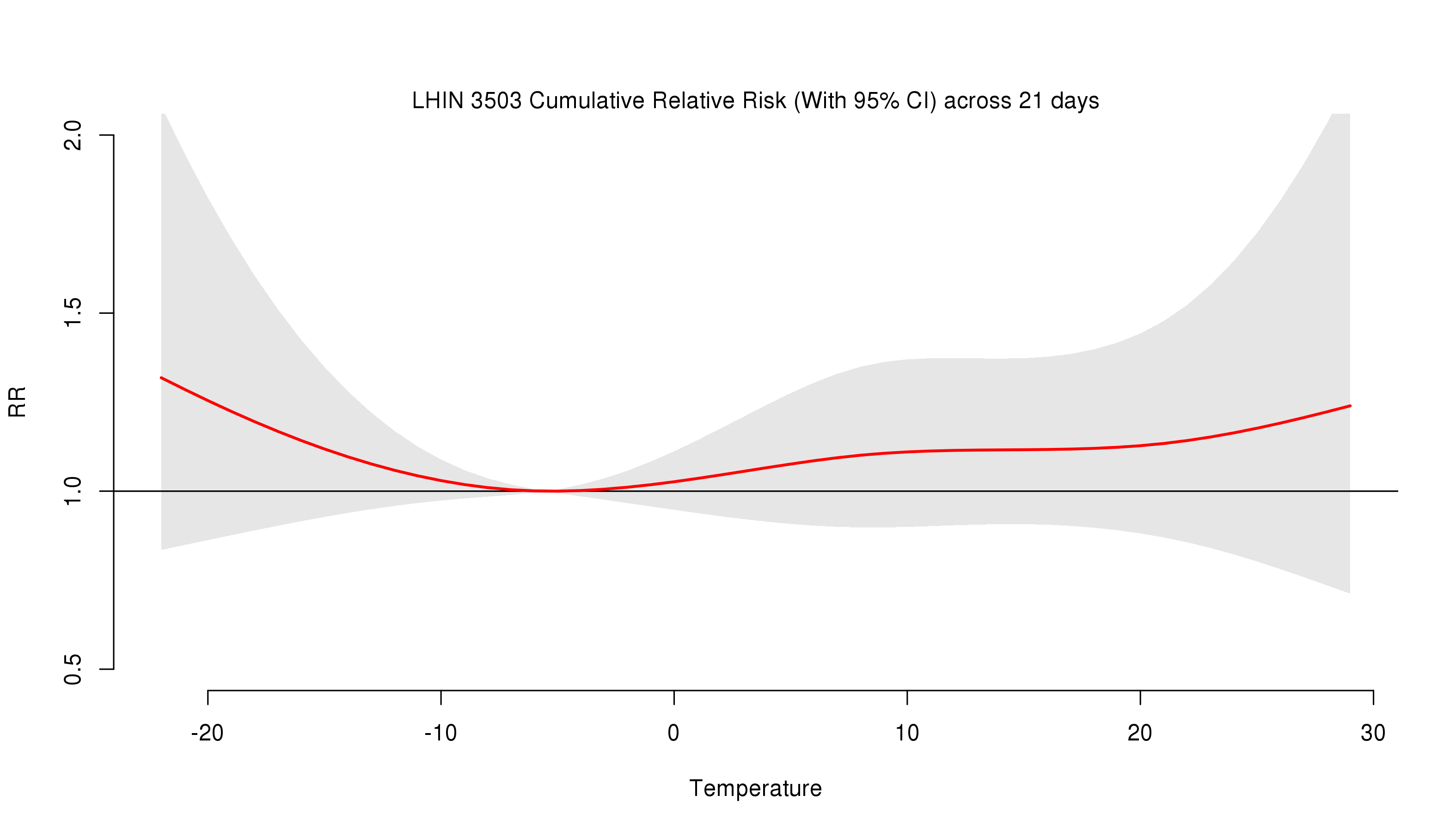

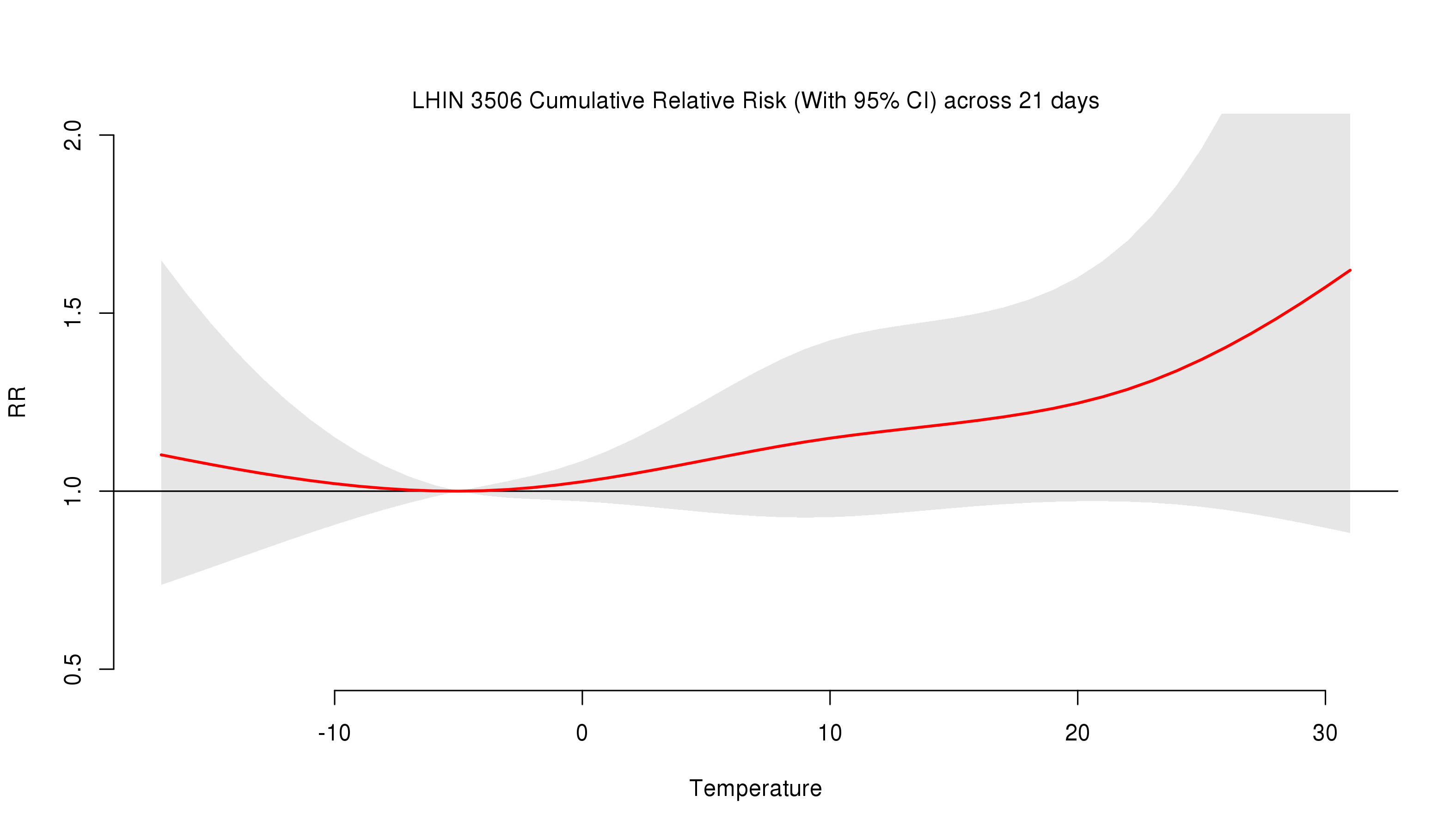


**(a)**

**(b)**


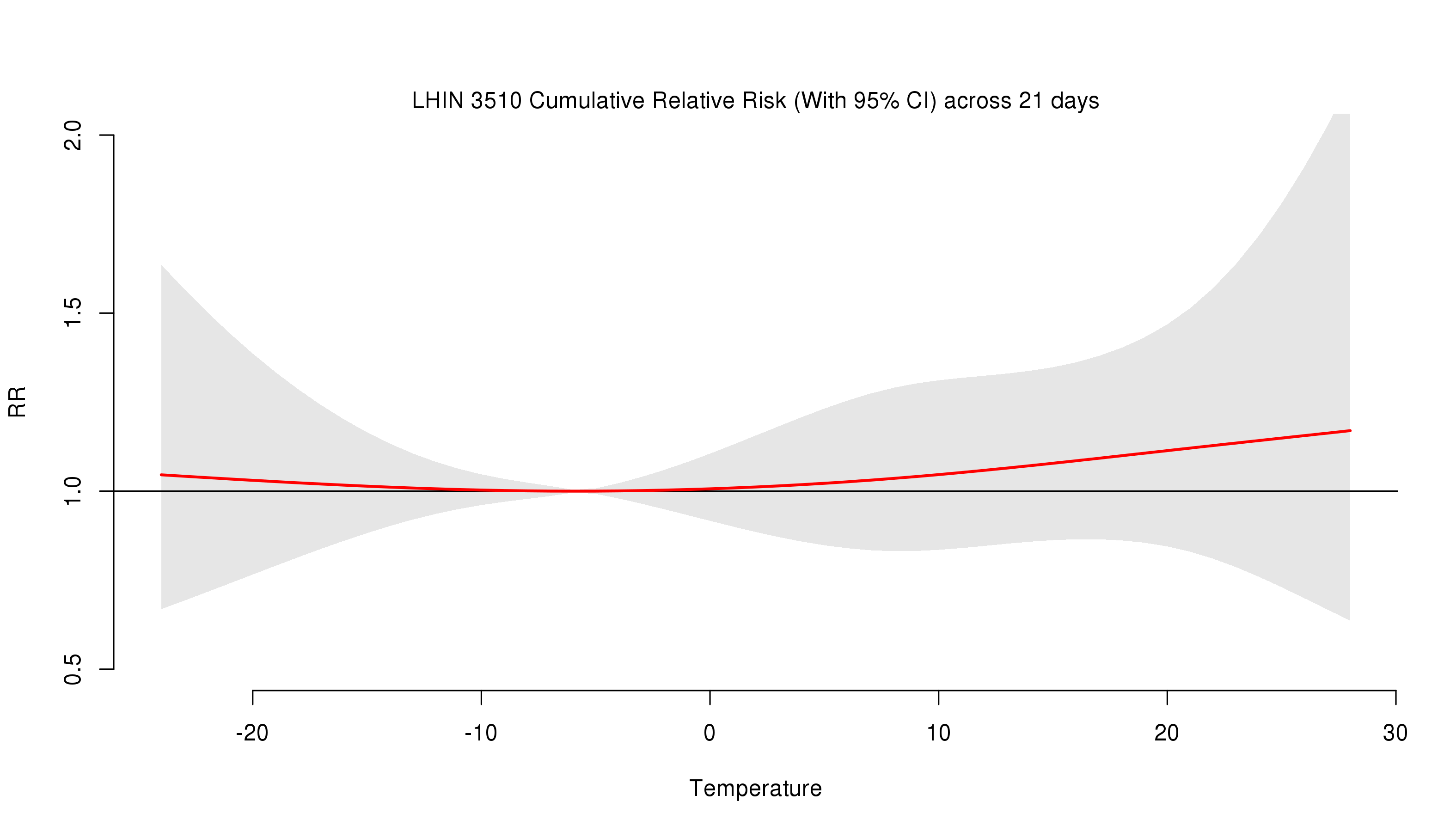

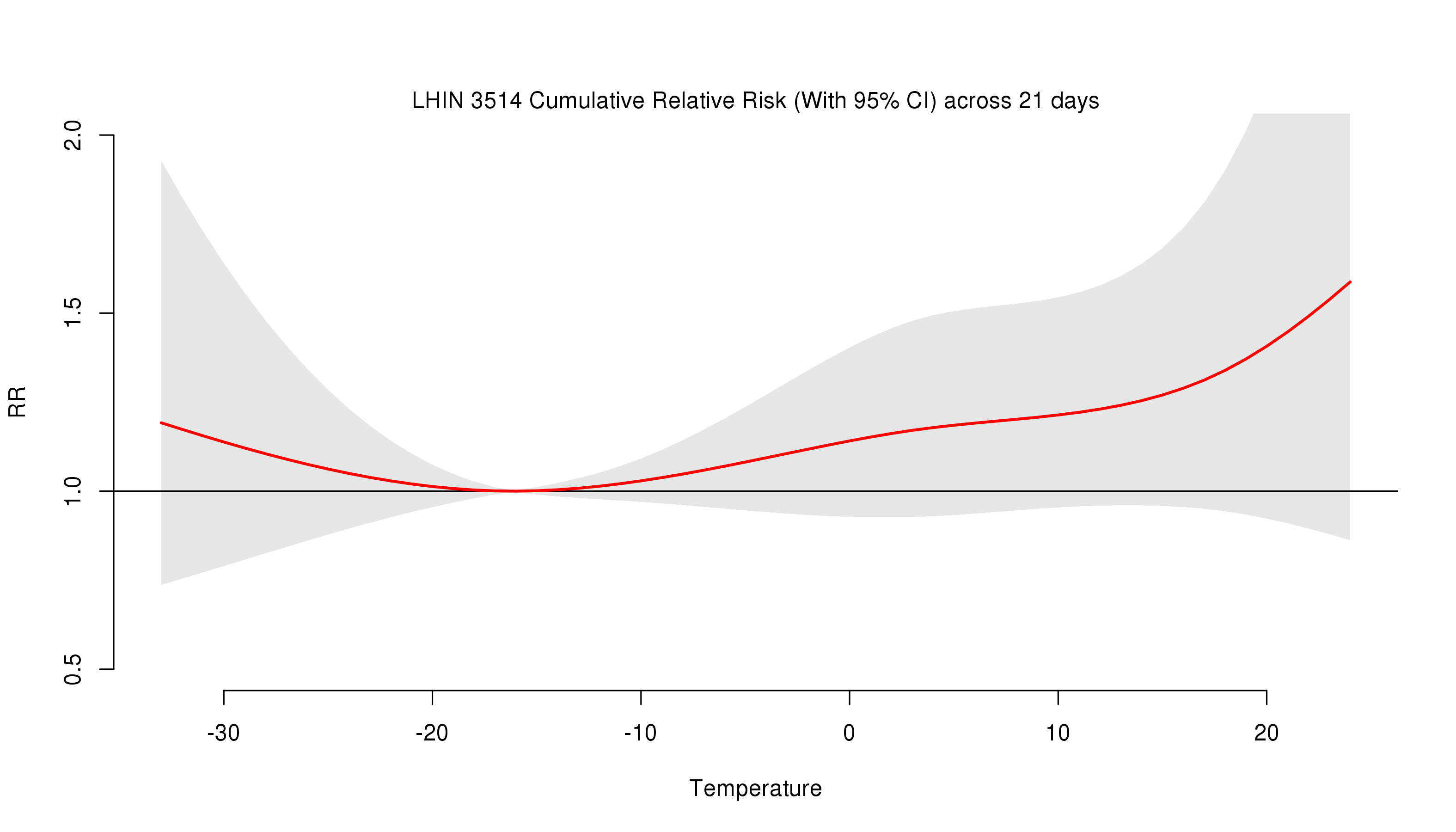


**(c)**

**(d)**

**Figure S2. Cumulative exposure–response associations of daily mean temperatures and daily hospital admissions for diabetes over a lag of 21 days in (a) Waterloo Wellington, (b) Mississauga Halton, (c) South East and (d) North West, 1996-2013.**


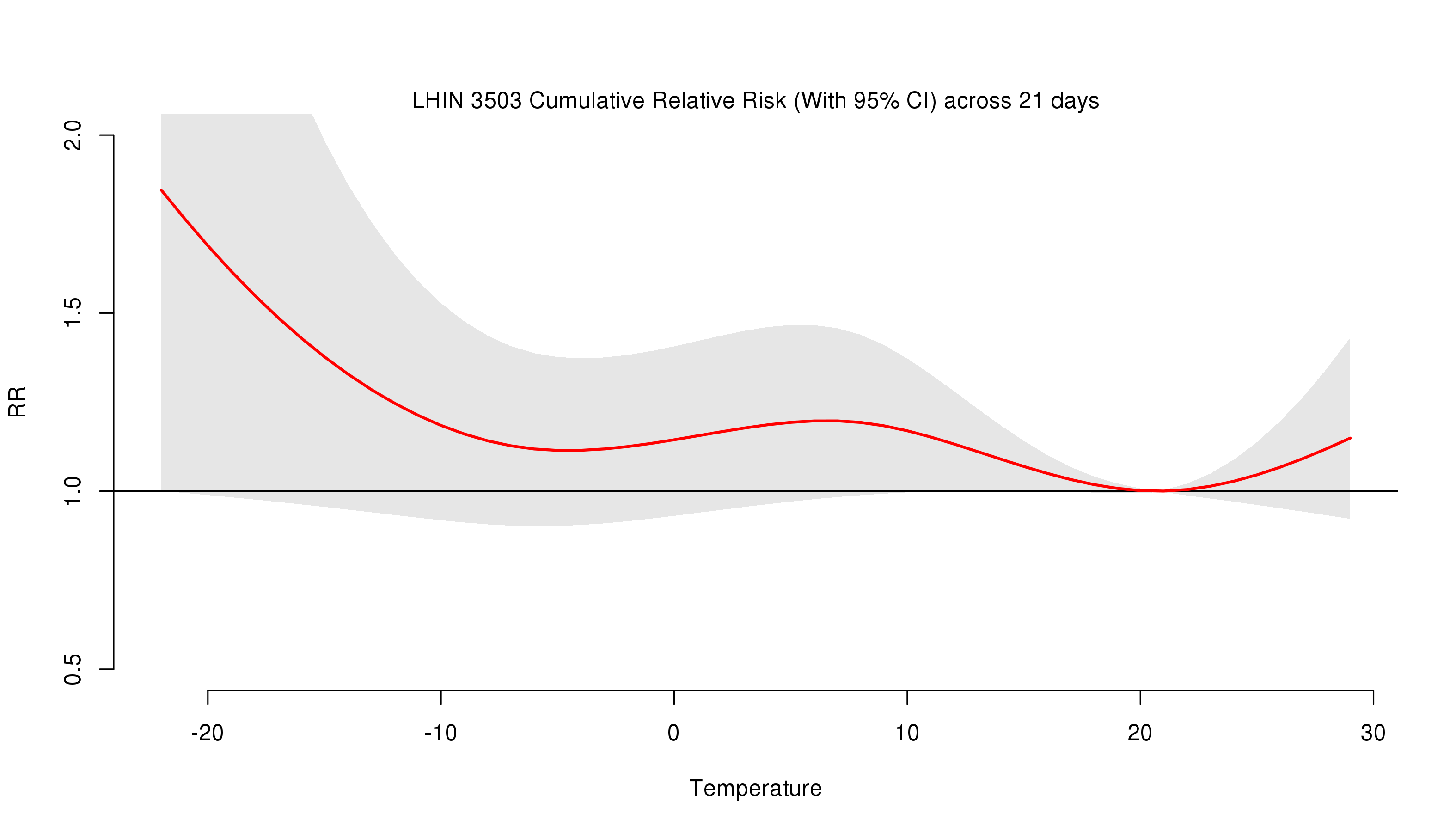

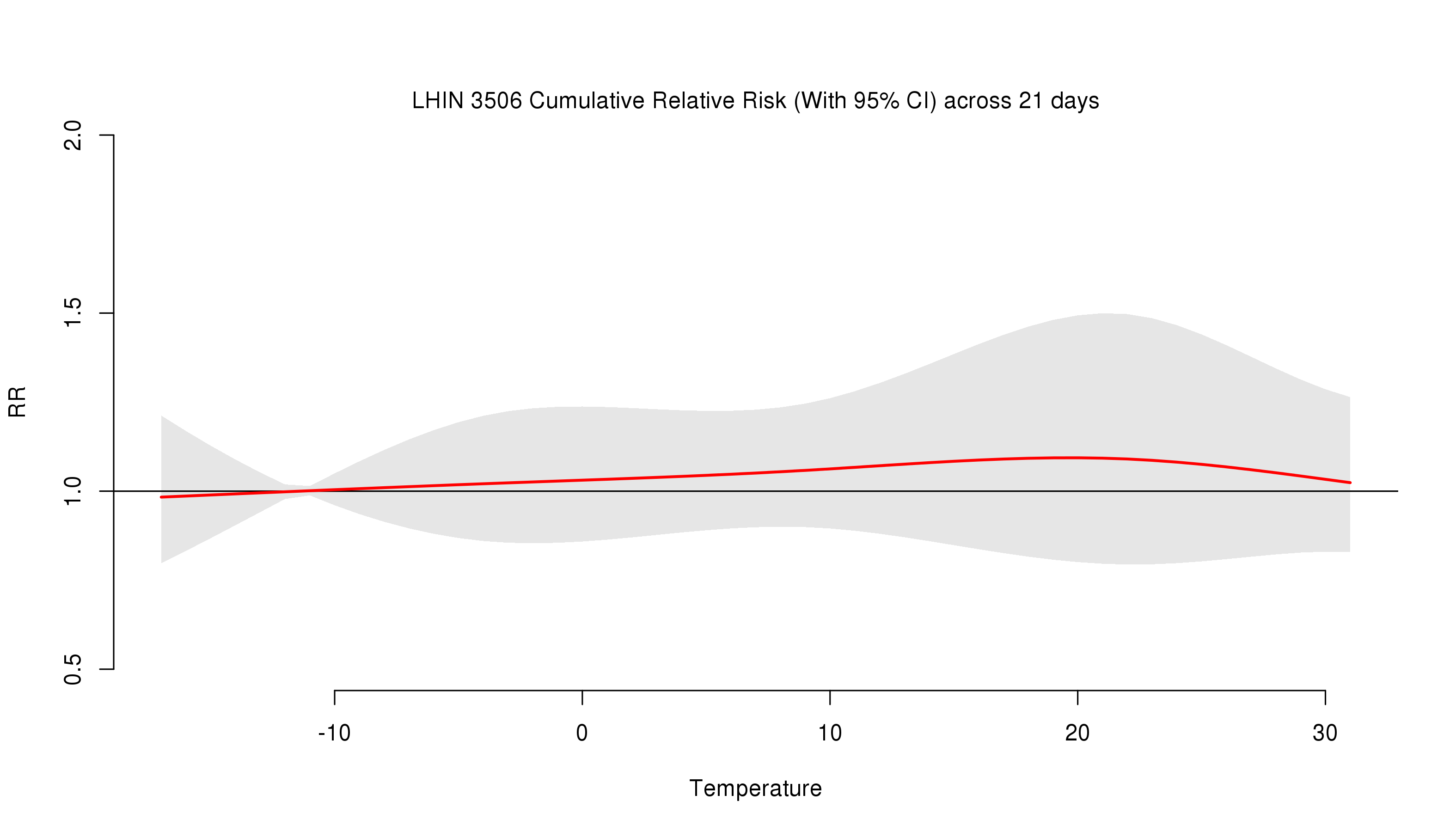


**(a)**

**(b)**


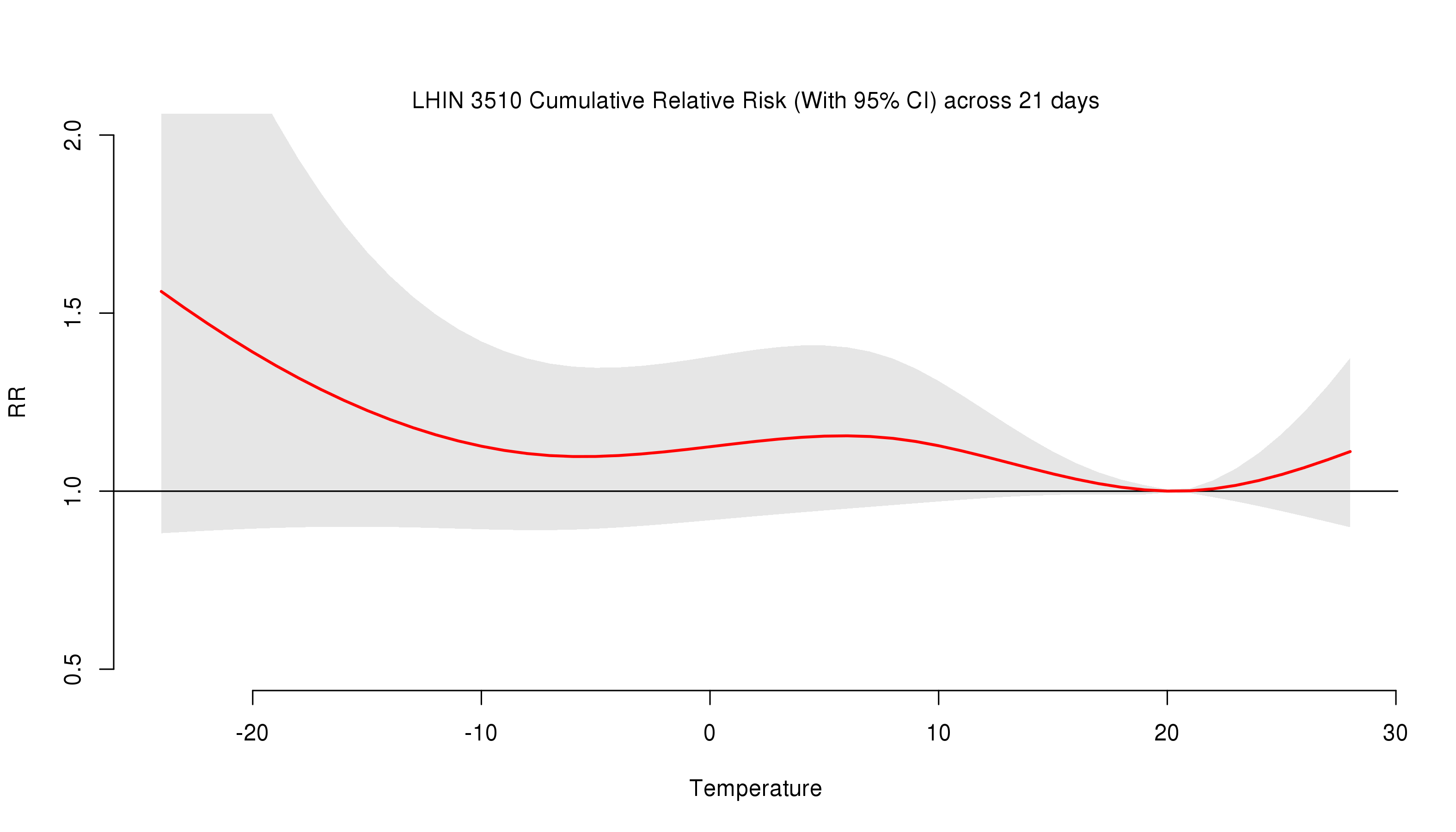

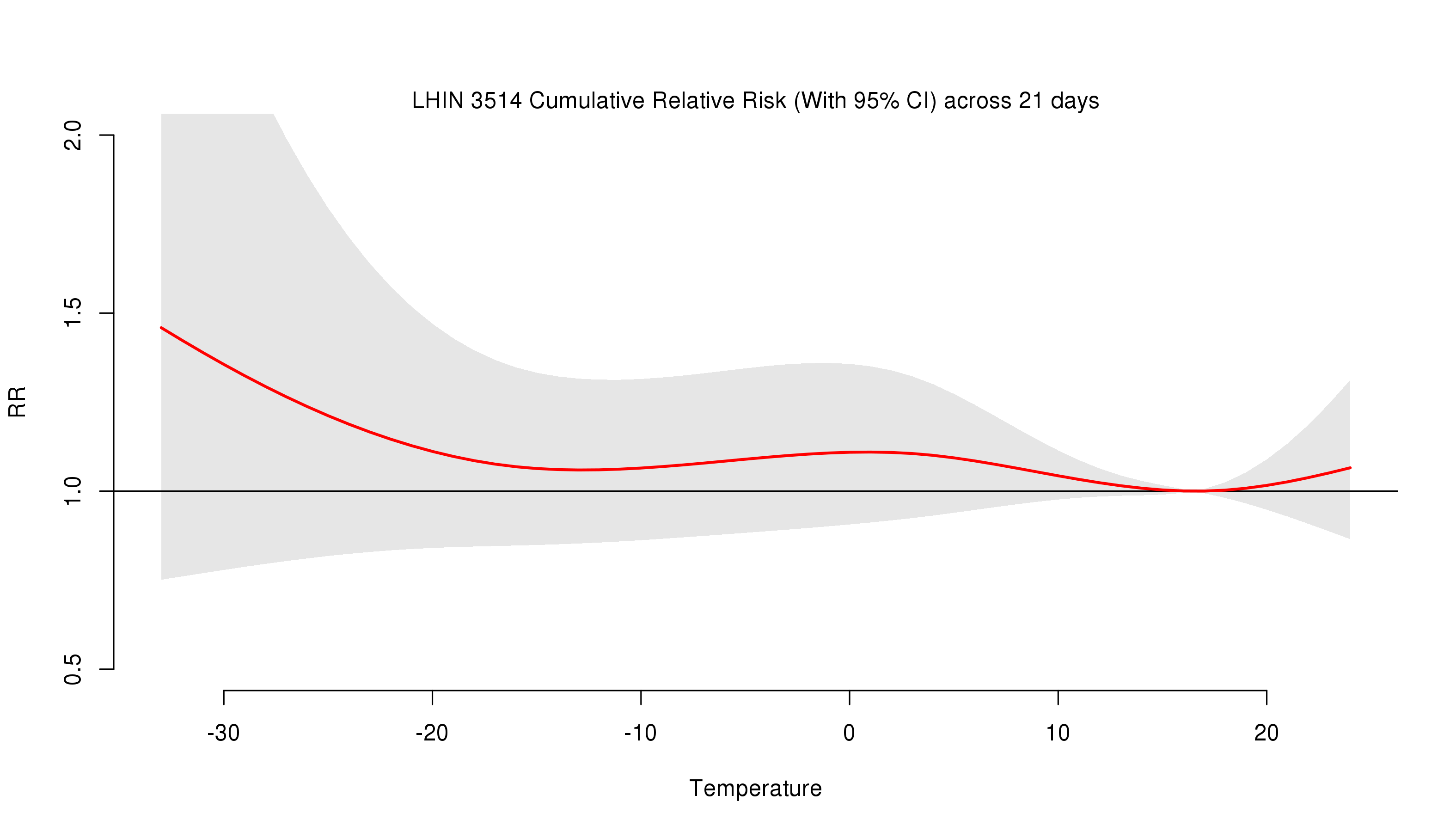


**(c)**

**(d)**

**Figure S3. Cumulative exposure–response associations of daily mean temperatures and daily hospital admissions for arrhythmia over a lag of 21 days in (a) Waterloo Wellington, (b) Mississauga Halton, (c) South East and (d) North West, 1996-2013**

**
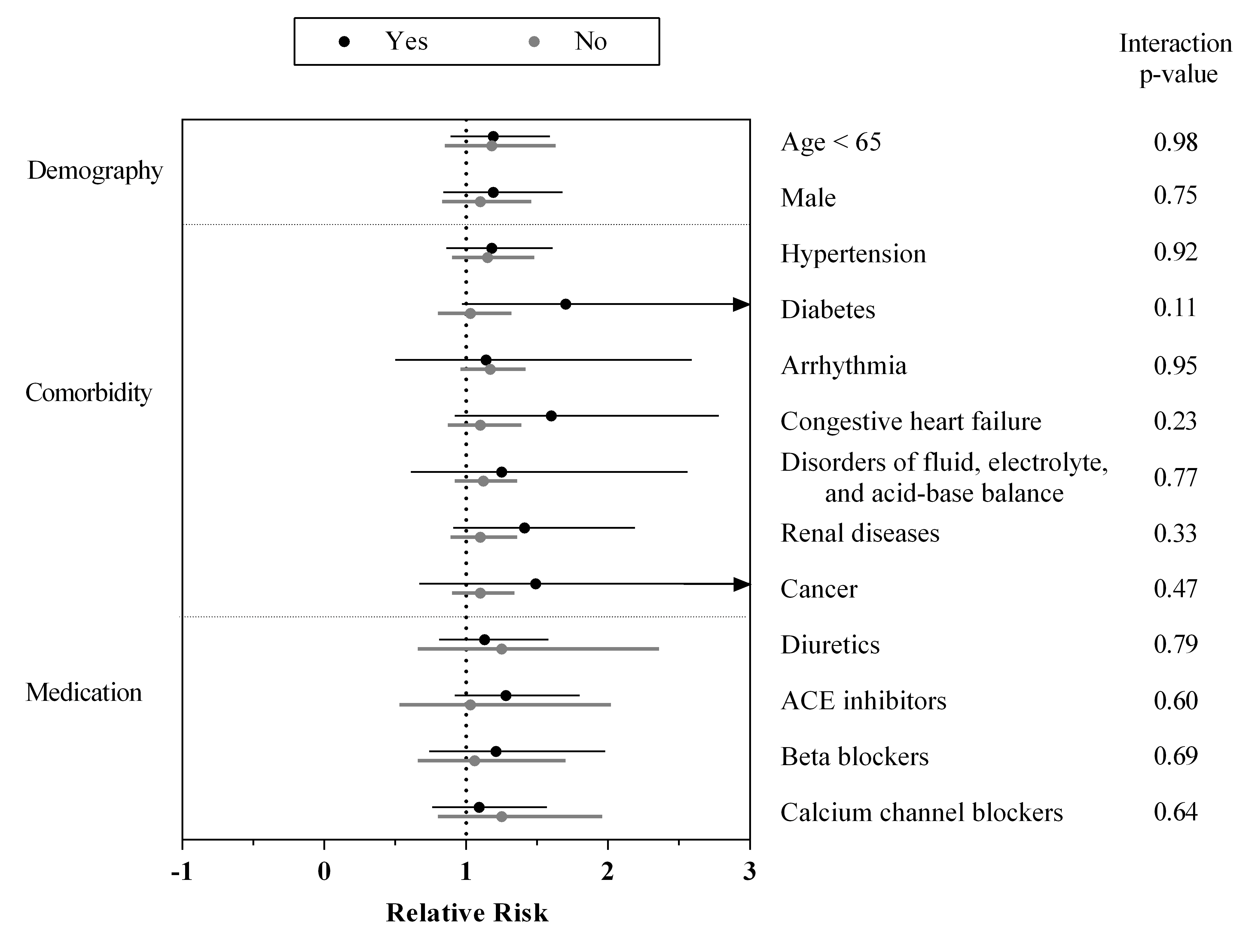
**

**Figure S4.** **Cumulative relative risks (RRs) and 95% confidence intervals (CIs) for the cold effects on daily hospitalizations for hypertension by individual characteristics, comorbid medical conditions and the intake of medication over 21 lag days in Ontario, Canada during 1996-2013.** Cold effects were examined by calculating relative risks associated with the 1^st^ percentile of temperature relative to the 25^th^ percentile of temperature. The subgroup analyses of medication intake were restricted to subjects aged 65 and over.

**
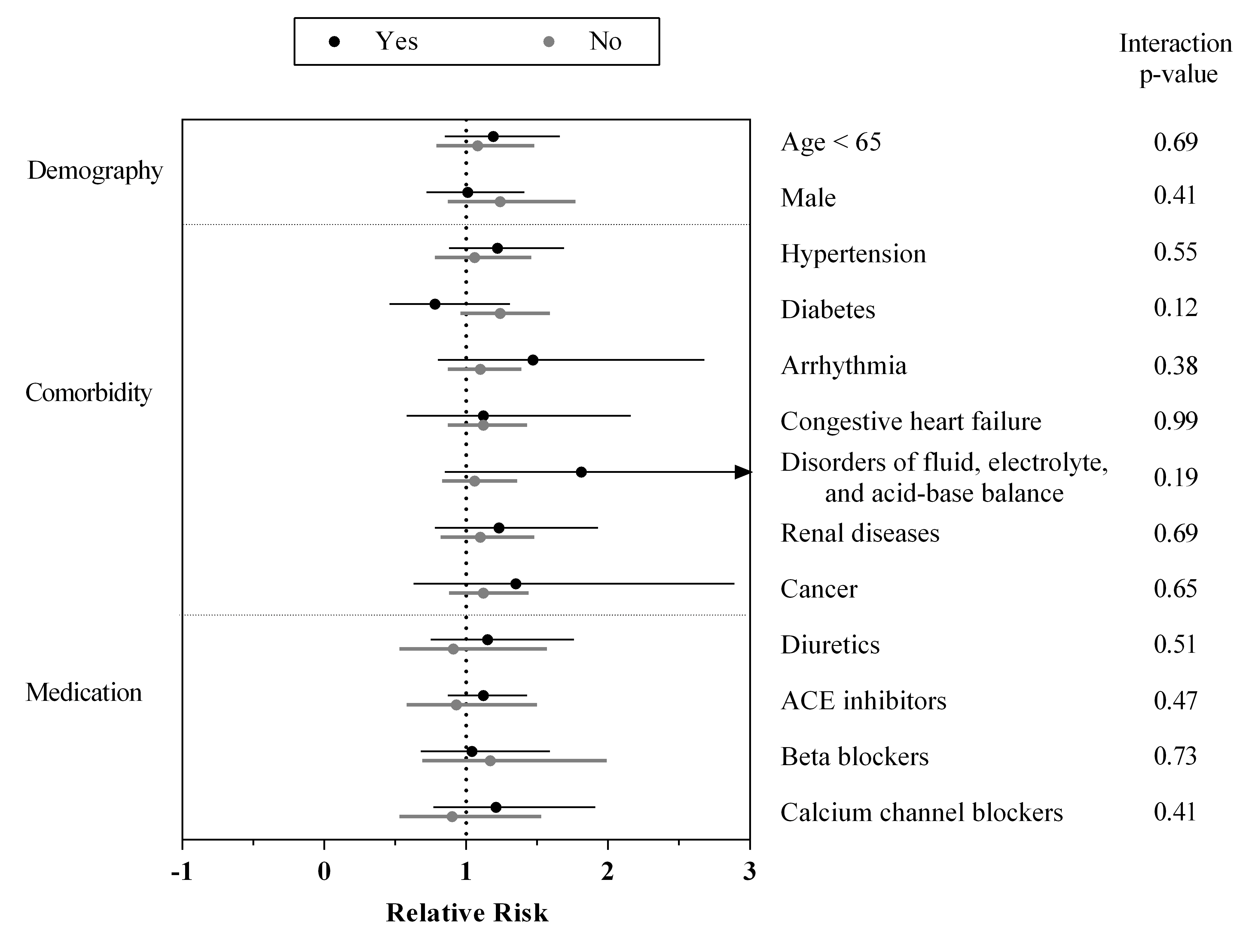
**

**Figure S5.** **Cumulative relative risks (RRs) and 95% confidence intervals (CIs) for the heat effects on daily hospitalizations for hypertension by individual characteristics, comorbid medical conditions and the intake of medication over 21 lag days in Ontario, Canada during 1996-2013.** Heat effects were examined by calculating relative risks associated with the 99^th^ percentile of temperature relative to the 75^th^ percentile of temperature. The subgroup analyses of medication intake were restricted to subjects aged 65 and over.

**
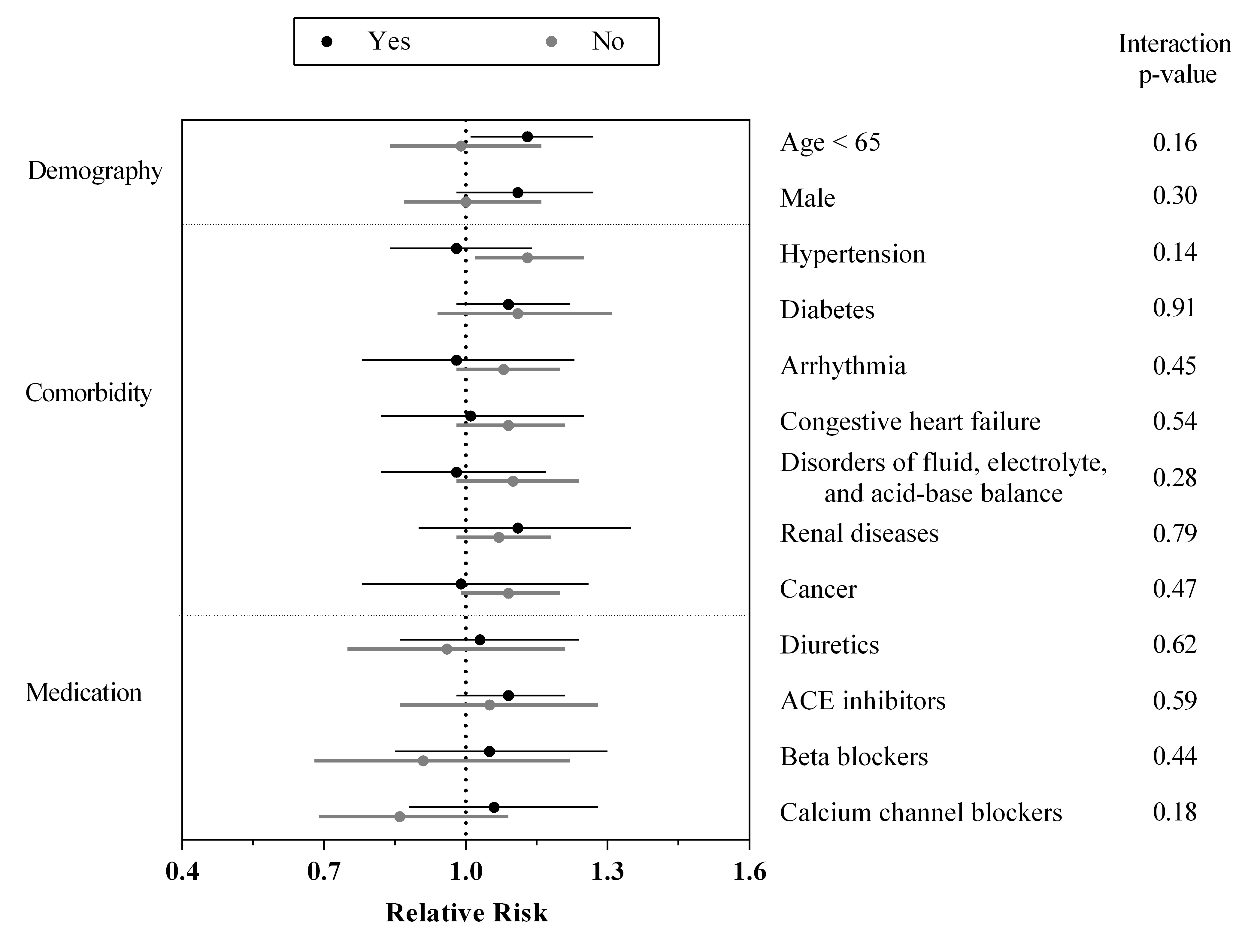
**

**Figure S6. Cumulative relative risks (RRs) and 95% confidence intervals (CIs) for the cold effects on daily hospitalizations for diabetes by individual characteristics, comorbid medical conditions and the intake of medication over 21 lag days in Ontario, Canada during 1996-2013.** Cold effects were examined by calculating relative risks associated with the 1^st^ percentile of temperature relative to the 25^th^ percentile of temperature. The subgroup analyses of medication intake were restricted to subjects aged 65 and over.

**
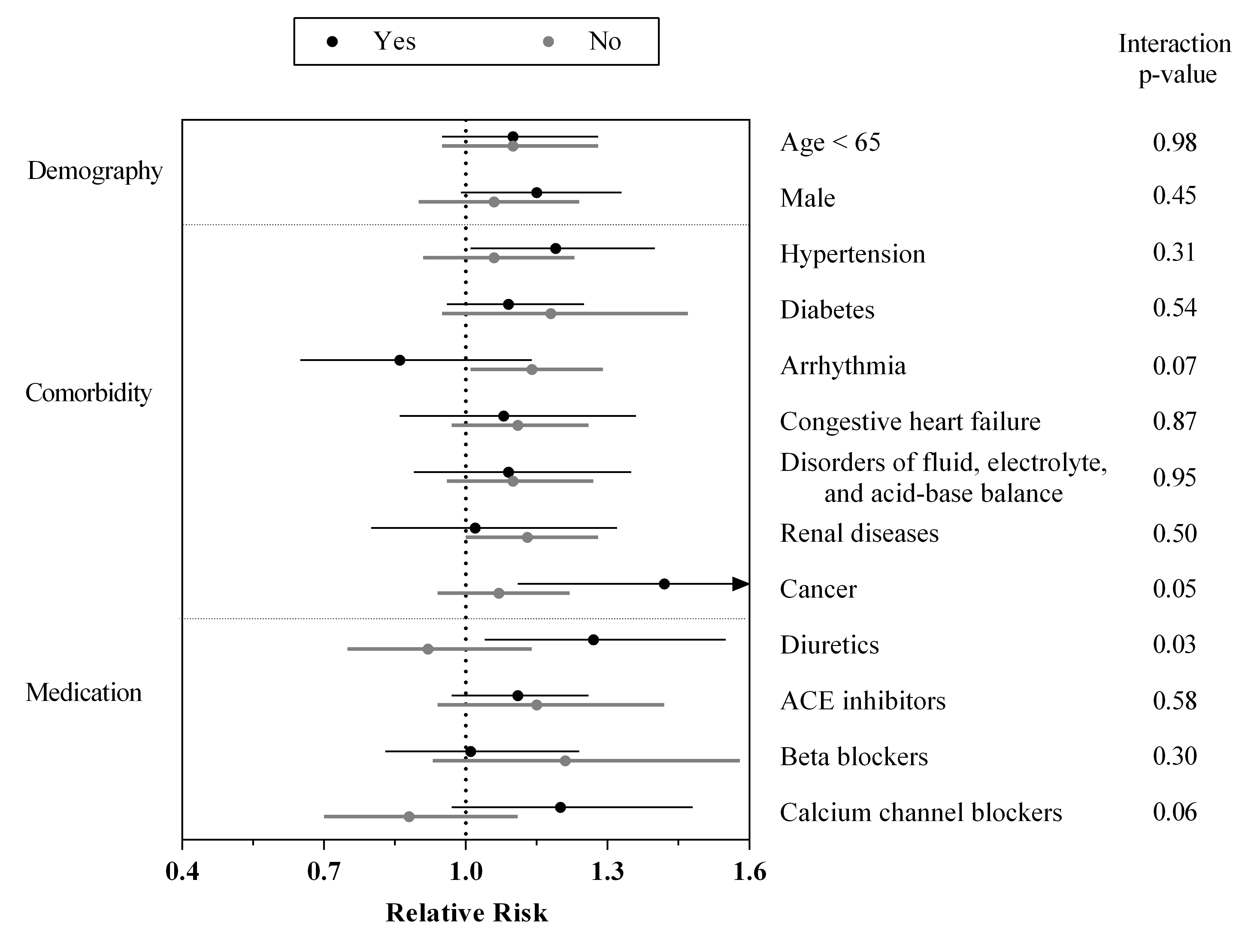
**

**Figure S7. Cumulative relative risks (RRs) and 95% confidence intervals (CIs) for the heat effects on daily hospitalizations for diabetes by individual characteristics, comorbid medical conditions and the intake of medication over 21 lag days in Ontario, Canada during 1996-2013.** Heat effects were examined by calculating relative risks associated with the 99^th^ percentile of temperature relative to the 75^th^ percentile of temperature. The subgroup analyses of medication intake were restricted to subjects aged 65 and over.

**
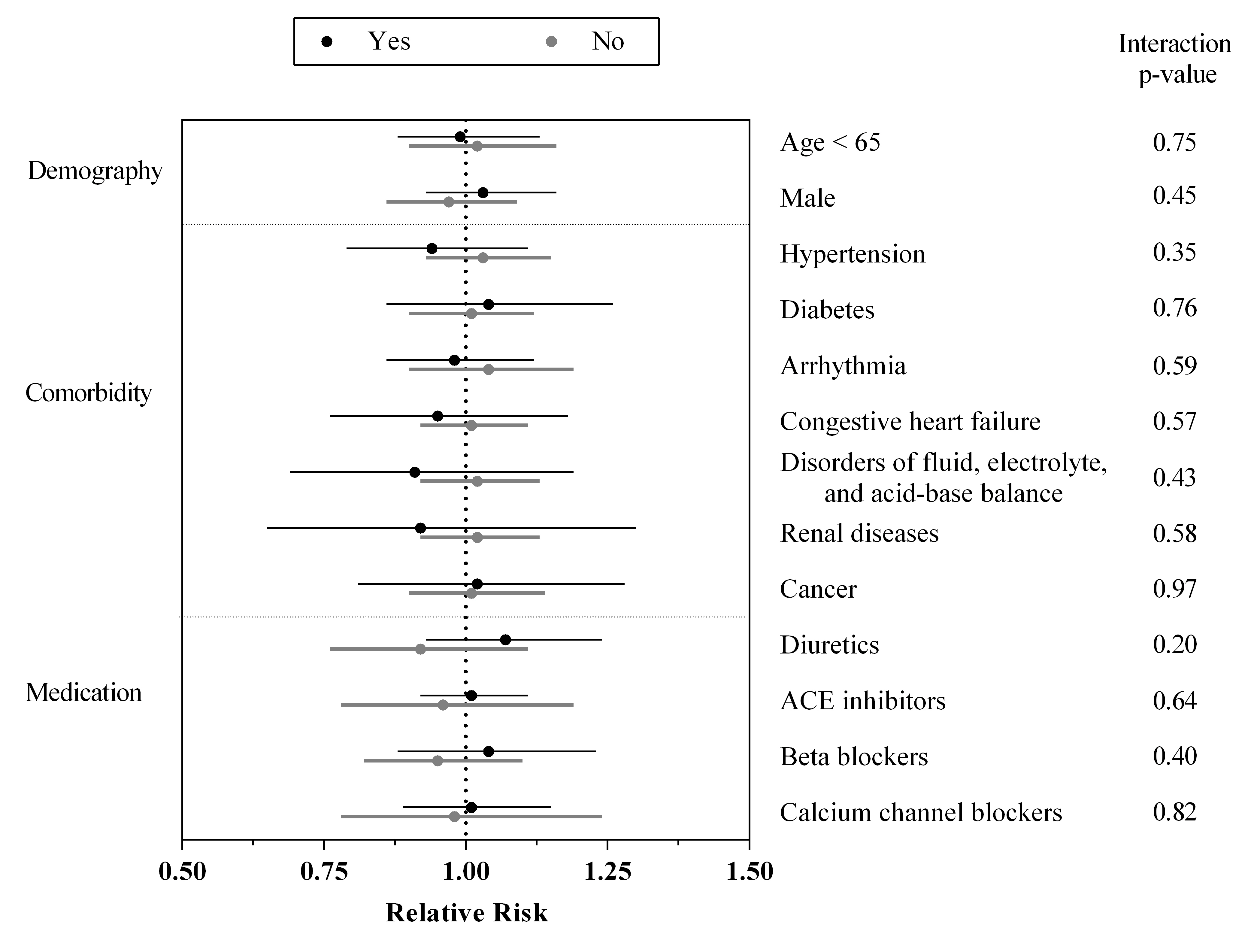
**

**Figure S8. Cumulative relative risks (RRs) and 95% confidence intervals (CIs) for the cold effects on daily hospitalizations for arrhythmia by individual characteristics, comorbid medical conditions and the intake of medication over 21 lag days in Ontario, Canada during 1996-2013.** Cold effects were examined by calculating relative risks associated with the 1^st^ percentile of temperature relative to the 25^th^ percentile of temperature. The subgroup analyses of medication intake were restricted to subjects aged 65 and over.

**
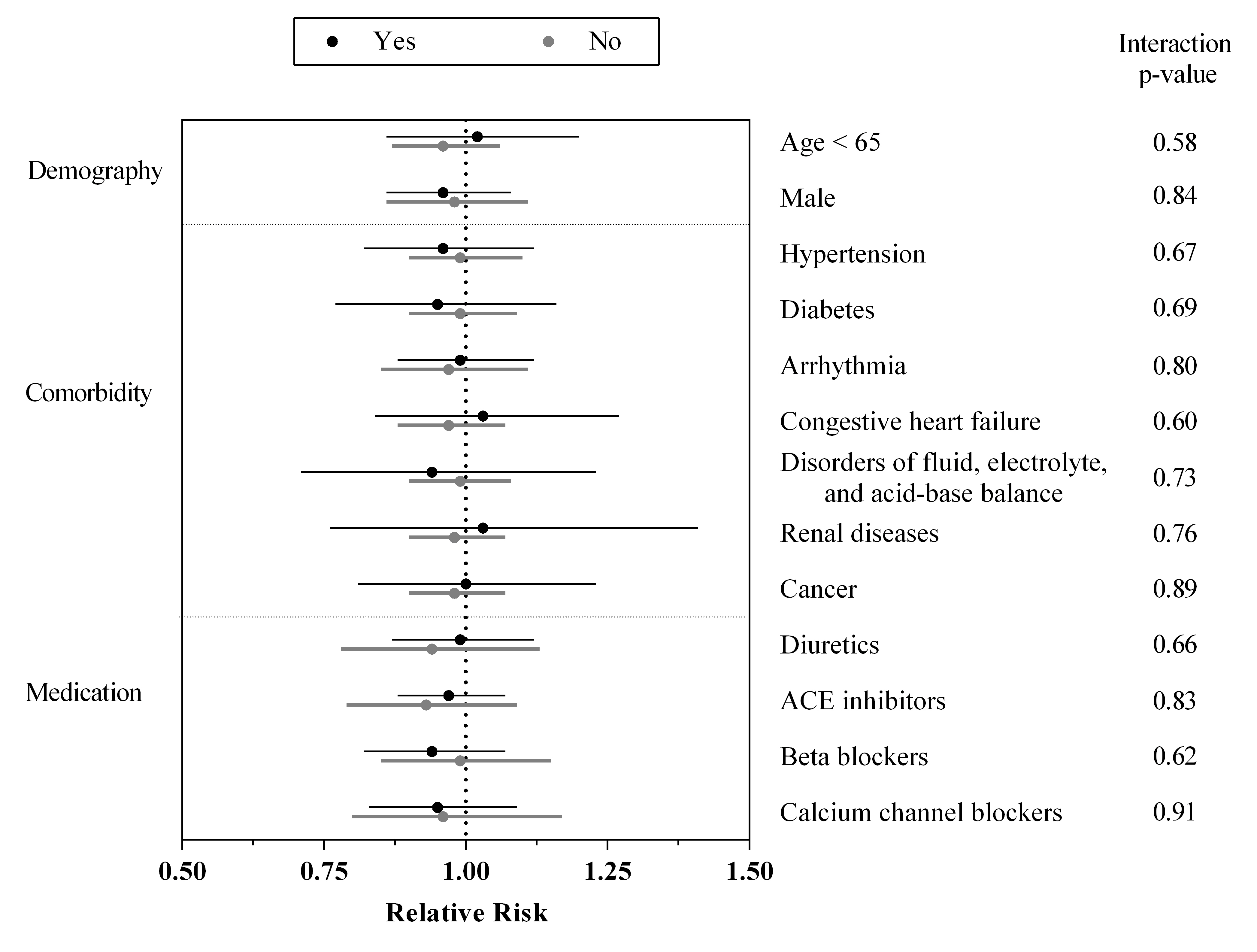
**

**Figure S9. Cumulative relative risks (RRs) and 95% confidence intervals (CIs) for the heat effects on daily hospitalizations for arrhythmia by individual characteristics, comorbid medical conditions and the intake of medication over 21 lag days in Ontario, Canada during 1996-2013.** Heat effects were examined by calculating relative risks associated with the 99^th^ percentile of temperature relative to the 75^th^ percentile of temperature. The subgroup analyses of medication intake were restricted to subjects aged 65 and over.


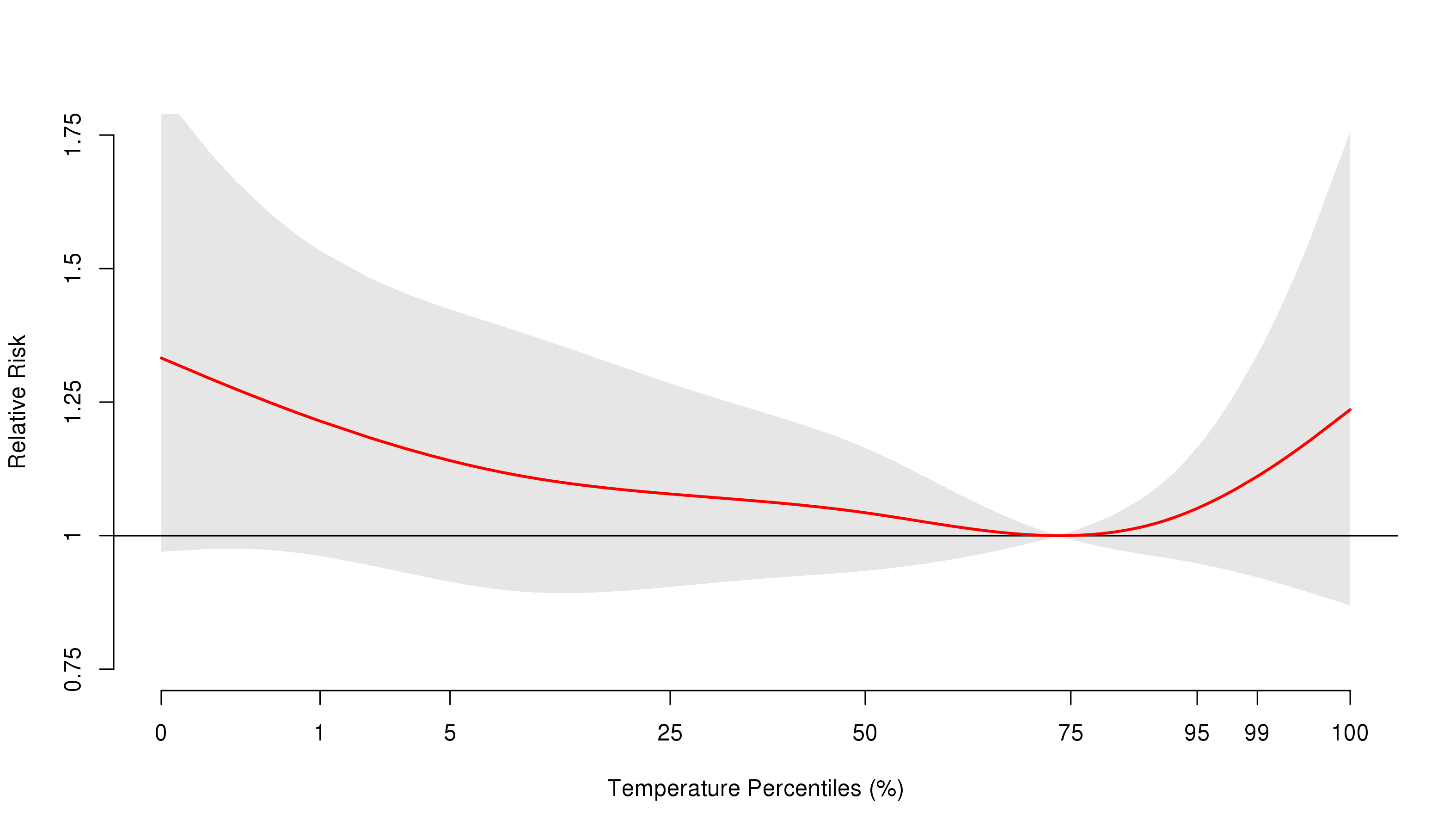


**(a)**


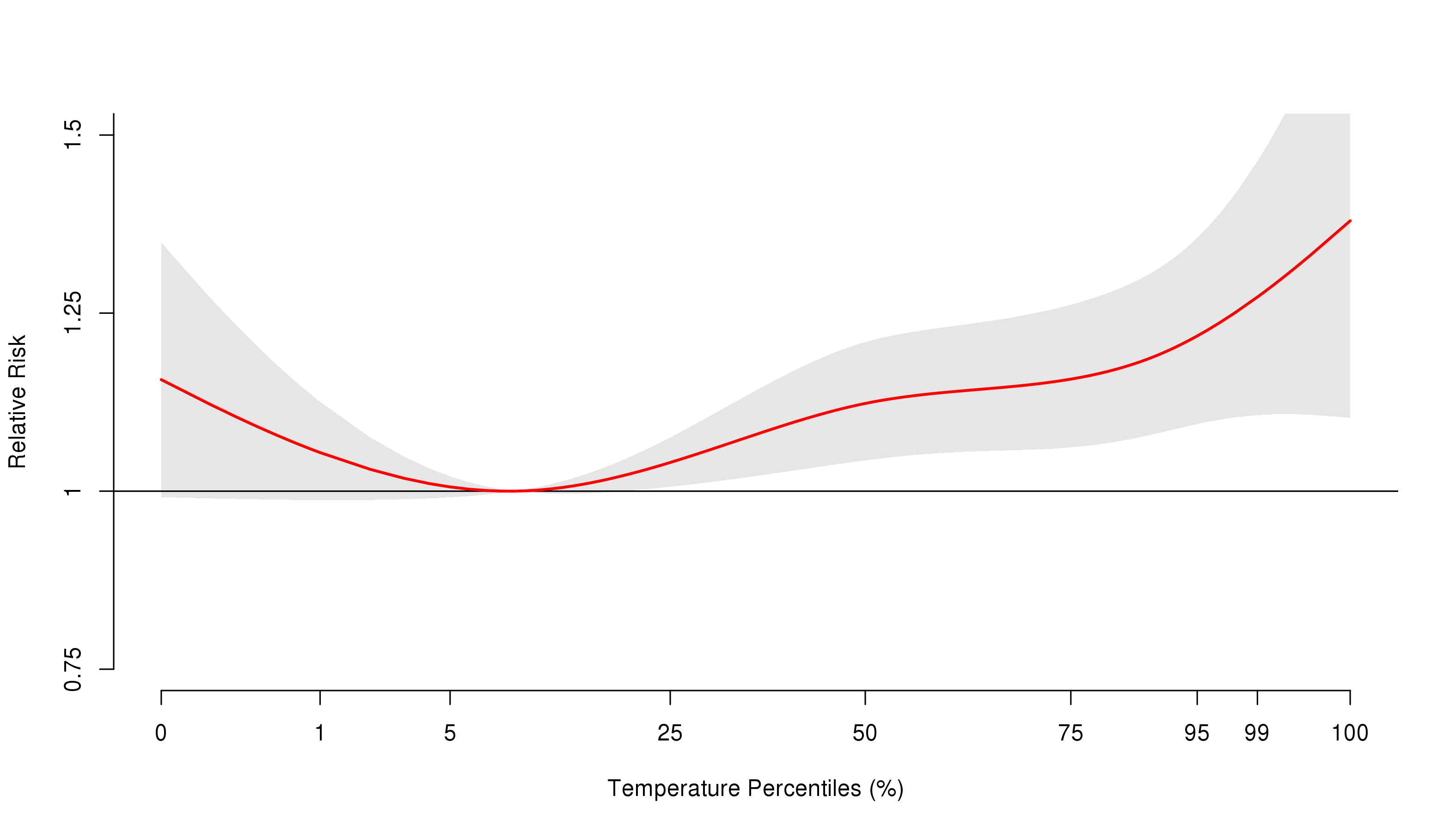


**(b)**


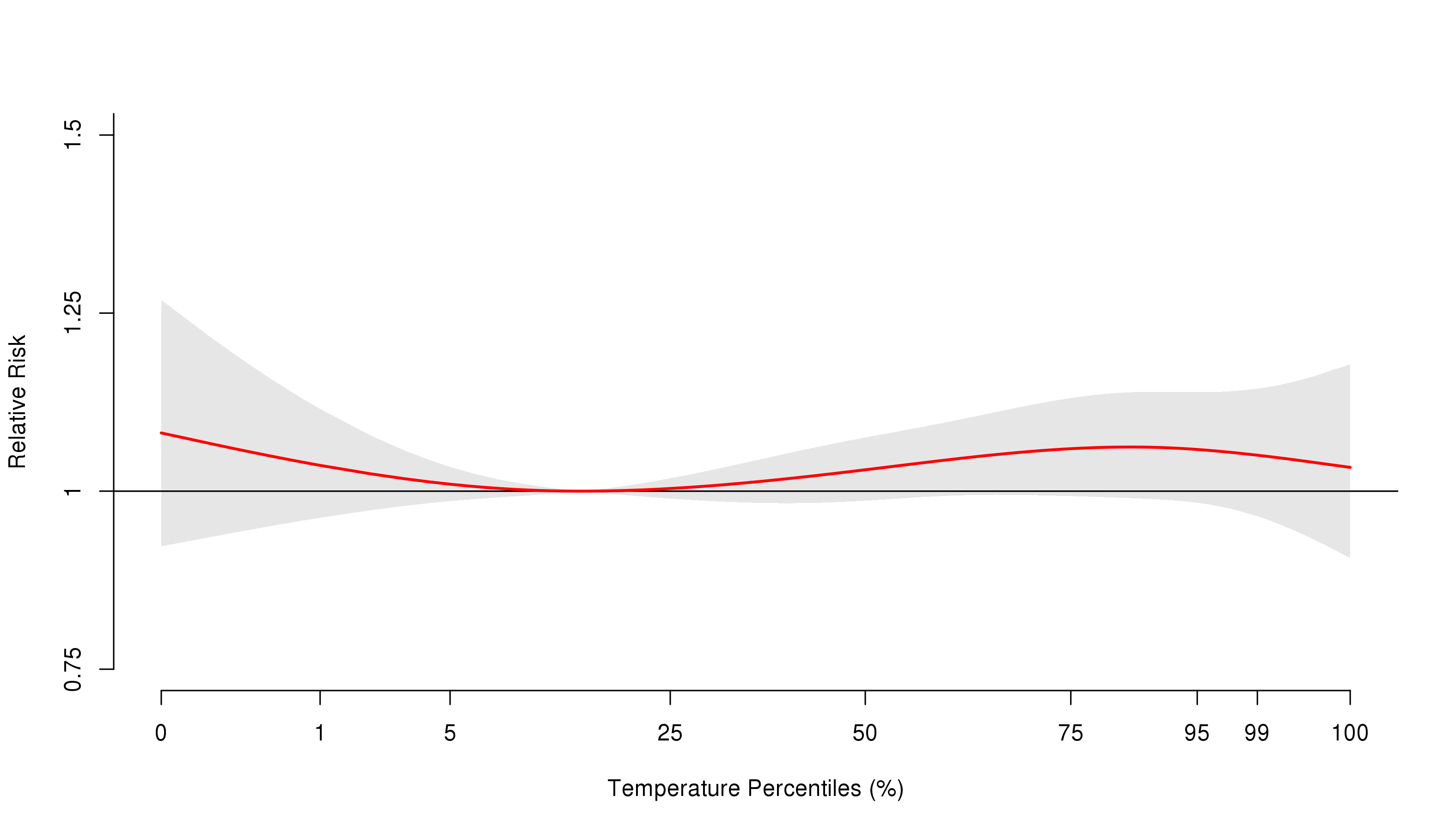


**(c)**

**Figure S10. Cumulative exposure–response associations of daily mean temperatures and daily hospital admissions for (a) hypertension, (b) diabetes, and (c) arrhythmia over a lag of 14 days in Ontario, Canada, 1996-2013**
